# TRPA1 aggravates osteoclastogenesis and osteoporosis through activating endoplasmic reticulum stress mediated by SRXN1

**DOI:** 10.1038/s41419-024-07018-5

**Published:** 2024-08-27

**Authors:** Pengfei Zhu, Huaqiang Tao, Kai Chen, Miao Chu, Qiufei Wang, Xing Yang, Jun Zhou, Huilin Yang, Dechun Geng

**Affiliations:** 1 Department of Orthopaedics, First Affiliated Hospital of Soochow University, Suzhou, 215006 Jiangsu China; 2 Department of Orthopedics, Hai’an People’s Hospital, Hai’an, 226600 Jiangsu China; 3 Department of Orthopedics, Yixing People’s Hospital, Yixing, 214200 Jiangsu China; 4 Department of Orthopedics, Changshu Hospital Affiliated to Soochow University, First People’s Hospital of Changshu City, Changshu, 215500 Jiangsu China; 5 Orthopedics and Sports Medicine Center, Suzhou Municipal Hospital, Nanjing Medical University Affiliated Suzhou Hospital, Suzhou, 215008 Jiangsu China

**Keywords:** Calcium and phosphate metabolic disorders, Molecular biology

## Abstract

Osteoporosis (OP) is a disorder of bone remodeling caused by an imbalance between bone resorption by osteoclasts and bone formation by osteoblasts. Therefore, inhibiting excessive osteoclast activity is one of the promising strategies for treating OP. A major transient receptor potential cation channel, known as transient receptor potential ankyrin 1 (TRPA1), was found to alleviate joint pain and cartilage degeneration in osteoarthritis. However, little research has focused on TRPA1 function in OP. As a result, this study aimed to explore the TRPA1 characteristics and its potential therapeutic function during osteoclastogenesis. The TRPA1 expression gradually increased in the osteoclast differentiation process; however, its suppression with small interfering RNA and an inhibitor (HC030031) significantly controlled the osteoclast count and the expression of osteoclast characteristic genes. Its suppression also inhibited endoplasmic reticulum (ER) stress-related pancreatic ER kinase (PERK) pathways. An ER stress inhibitor (thapsigargin) reversed the down-regulated levels of ER stress and osteoclast differentiation by suppressing TRPA1. Transcriptome sequencing results demonstrated that TRPA1 negatively regulated reactive oxygen species (ROS) and significantly increased the expression of an antioxidant gene, SRXN1. The osteoclast differentiation and the levels of ER stress were enhanced with SRXN1 inhibition. Finally, TRPA1 knockdown targeting macrophages by adeno-associated virus-9 could relieve osteoclast differentiation and osteopenia in ovariectomized mice. In summary, silencing TRPA1 restrained osteoclast differentiation through ROS-mediated down-regulation of ER stress via inhibiting PERK pathways. The study also indicated that TRPA1 might become a prospective treatment target for OP.

## Introduction

In elderly individuals, osteoporosis (OP) is the most common metabolic bone disease, characterized by loss of bone mass and trabecular breakdown [1]. This makes the skeleton more fragile and increases the likelihood of fractures. Current OP treatments include antiresorptive agents and osteoanabolic agents, such as biphosphonates and parathyroid hormone [2]. However, long-term therapy could lead to secondary effects, including an increased risk of thromboembolic disease and atypical femur fracture [3, 4]. Given the increasing prevalence of OP, further in-depth studies of the mechanism are necessary for effective treatment.

Osteoclasts are multinucleated cells of the bone marrow lineage responsible for bone resorption through matrix degradation. Osteoclast formation is significantly regulated by the production of cytokine receptor activator for nuclear factor-κB ligand (RANKL) through osteoblasts and osteoclasts [5]. The binding of RANKL with receptor activator of nuclear factor kappa-B activates nuclear factor kappa-B, nuclear factor-activated T cells c1 (NFATc1), and other signaling pathways that control the expression of osteoclast-related genes, such as matrix metalloproteinase-9 (MMP9) and tartrate-resistant acid phosphatase (TRAP) [6, 7]. During bone remodeling, excessive osteoclast activation could cause bone resorption to exceed bone formation and osteopenia. Therefore, one of the most promising therapies for OP is inhibiting osteoclast-mediated bone resorption.

As nonselective cation channels, transient receptor potential (TRP) triggers Ca^2+^ influx upon activation, playing a critical role in numerous signaling pathways regulating cell survival, proliferation, and homeostasis. Transient receptor potential ankyrin 1 (TRPA1) is a nonselective calcium-permeable TRP family channel. The primary function of TRPA1 is to detect a wide range of exogenous stimuli capable of damaging cells. It plays a crucial role in inflammation, endoplasmic reticulum (ER), and oxidative stress in intervertebral disc degeneration [8], respiratory diseases [9], and cardiovascular disorders [10]. The blocking of Ca^2+^ influx and ER or mitochondrial oxidative stress by TRPA1 antagonists has been observed to significantly reduce the production of reactive oxygen species (ROS) and hypoxia-induced apoptosis. TRPA1 has been found to alleviate acute inflammation, joint pain, and cartilage degeneration in osteoarthritis [11]. Previous research revealed that TRPA1 inhibition significantly reduced cardiomyocyte apoptosis caused by doxorubicin by alleviating ER stress [12]. Deng et al. also found that TRPA1 promotes mitochondrial morphology and dysfunction in cisplatin-induced renal tubular epithelium cell damage by inducing ER stress [13]. These findings indicate that TRPA1 regulates ER stress, making it an attractive target for treating clinical disorders in various pathological processes.

ER stress occurs due to the accumulation of unfolded proteins, influenced by various factors such as starvation and oxidative stress [14]. For reducing unfolded proteins and recovering protein homeostasis, the unfolded protein response (UPR), involving pancreatic ER kinase (PERK), inositol-requiring enzyme 1α (IRE1α), and activating transcription factor 6, is activated in mammalian cells [15]. Recent research indicated that silencing PERK alleviated osteopenia in a mouse model of OP by inhibiting oxidative stress-mediated ER stress [16]. Another study revealed that the IRE1α pathway induced by ER stress is an important regulator for accelerating osteoclast differentiation by promoting NFATc1 transcription [17]. Furthermore, preliminary studies suggested that the levels of ER stress increased during osteoclastogenesis. Although ER stress is strongly associated with osteoclast differentiation and TRPA1, there is limited research focusing on the function of TRPA1 on ER stress in osteoclasts.

Based on the current research, we discovered the activation of TRPA1 in ovariectomy (OVX) mice and RAW 264.7 cells. Furthermore, inhibition of TRPA1 suppressed ER stress, decreased osteoclast formation, and alleviated bone loss in vivo. In terms of mechanism, TRPA1 accelerates osteoclast differentiation by promoting ER stress mediated by ROS. Our results suggested that suppressing ER stress could ameliorate osteoclastogenesis, and TRPA1 may hold promise as an attractive therapeutic target for OP.

## Materials and methods

### Reagents

Small interfering RNAs (Si-RNA) targeting TRPA1 and SRXN1 were acquired from GenePharma (Suzhou, China). Their sequences are listed in Table 1. HC030031 was obtained from MedChemExpress (New Jersey, USA), and dimethyl sulfoxide (Sigma-Aldrich, St. Louis, USA) was used as the solvent for its dilution. Recombinant RANKL was obtained from R and D Systems (Minneapolis, USA). The adeno-associated virus-9 (AAV9) targeting TRPA1 was constructed by Hanbio Tech (Shanghai, China). The sequences as follows: 5-3, sense, CCUAGGAUCUUAUUGUCUUTT; anti-sense, AAGACAAUAAGAUCCUAGGTT.

**Table 1:** Sequences of Si-RNA targeting TRPA1 and SRXN1.

TRPA1	Si-710	Sense Anti-sense	GCAGGGAGACUCACAUUAATT UUAAUGUGAGUCUCCCUGCTT
	Si-1091	Sense Anti-sense	CCUGGAACAUUGUGAAUUUTT AAAUUCACAAUGUUCCAGGTT
	Si-2199	Sense Anti-sense	CCUAGGAUCUUAUUGUCUUTT AAGACAAUAAGAUCCUAGGTT
SRXN1	Si-447	Sense Anti-sense	CCAUCGACGUCCUCUGGAUTT AUCCAGAGGACGUCGAUGGTT
	Si-478	Sense Anti-sense	GGGUGGCGACUACUACUAUTT AUAGUAGUAGUCGCCACCCTT
	Si-603	Sense Anti-sense	GAGCAUCCACACCAGACUUTT AAGUCUGGUGUGGAUGCUCTT

### Cell culture

Under corresponding treatment conditions, RAW 264.7 macrophages were used to induce osteoclast differentiation. The Cell Bank of the Chinese Academy of Sciences (Shanghai, China) provided RAW 264.7 cells. Dulbecco’s modified eagle medium (DMEM), which contains high glucose, was used as the growth medium. For experimental purposes, the cells were collected and utilized.

### OVX-induced OP mouse model

All animal experiments were approved by the Ethics Committee of the First Affiliated Hospital of Soochow University. We randomly divided 21 female C57BL/6 mice (six weeks old) into three groups (*n* = 7 in each group): the Sham group, the OVX group, and the AAV9-TRPA1 group. For the surgery, we made an incision along the midline of the back and separated the muscle tissues to assess the ovaries under anesthesia. The ovaries were then excised after ligation of the oviduct. To prevent infection, penicillin was administered three days after the operation. One week after OVX, injection of AAV9-TRPA1 into the marrow cavity was carried out at the epiphyseal transition of the femur through the periosteum and cortex with a single dose of virus (2 μL) containing 2 × 10^9^ AAV9-TRPA1 vector genomes once every month. All the animals were kept and fed in a constant temperature and humid environment. We extracted samples of femurs for micro-CT and histology detections two months after surgery.

### Micro-CT analysis

An excess dose of pentobarbital (Sigma-Aldrich, USA) was administered to euthanize all experimental mice. We assessed the microarchitecture of bone tissues using a high-resolution micro-CT system (SkyScan1176, Aartselaar, Belgium) at a high resolution (9 µm) and a 50 kV beam energy. Micro-CT analysis was performed on the trabecular bone 1 mm beneath the growth plate of the distal femur. A systematic evaluation and calculation of bone mineral density (BMD, g/cm^3^), bone volume ratio (BV/TV, %), trabecular number (Tb. N, 1/mm), and trabecular separation (Tb. Sp, mm) was conducted.

### Bone morphological analysis

The femurs were fixed in formalin and decalcified in ethylene-diamine tetraacetic acid (Sigma-Aldrich, USA) for three weeks after scanning. After embedding in paraffin, the tissues were sliced into 6-µm-thick sections. Following previous protocols, hematoxylin and eosin staining and TRAcP activity staining were performed [18]. Osteoclasts were identified as cells staining red with a distinct border. Images were captured using an Axiovert 40C optical microscope (Zeiss, Jena, Germany).

### Histological immunofluorescence staining

After deparaffinization and antigen retrieval, primary antibodies against NFATc1 (Abclonal, A24872) and TRPA1 (Abclonal, A8568) were added for incubation at 4 °C. Following rinsing with PBS, the sections were incubated in the dark with the fluorescent secondary antibody. All sections were counterstained with DAPI (Beyotime, Suzhou, China). ImageJ software (Media Cybernetics, Bethesda, USA) was employed to assess the fluorescence intensity.

### Osteoclastogenesis assay

RAW 264.7 cells were cultured in a 24-well plate with a density of 3 × 10^4^ and added with 50 ng/mL RANKL. Until osteoclastic differentiation occurred, the medium was replaced every two days and continued until osteoclast formation. After fixation with paraformaldehyde (4 wt%, Biosharp, Guangzhou, China), the TRAcP kit (Bizhong Bio, Suzhou, China) was used to detect osteoclasts. TRAcP^+^ cells with three or more nuclei and sharp outlines were distinguished as osteoclasts. Under an inverted microscope (Zeiss, Germany), images were collected and counted at a specific magnification.

### Western blot analysis

Cellular proteins were extracted from different groups using RIPA buffer (Beyotime, China). Proteins were separated by SDS-PAGE and transferred onto PVDF membranes (Beyotime, China). After extracting with RIPA buffer (Beyotime, China), the proteins separated from SDS-PAGE were transferred to PVDF membranes (Beyotime, China). Primary antibodies against MMP9 (Abclonal, A0289), NFATc1 (Abclonal, A24872), TRPA1 (Abclonal, A8568), PERK (Abclonal, A18196), p-PERK (Abclonal, AP0086), eIF2α (Abclonal, A0764), p-eIF2α (Abclonal, AP0745), ATF4 (Abclonal, A18687), CHOP (Abclonal, A0221), and β-actin (Abclonal, AC006), together with a secondary antibody (Beyotime, China), were added to incubate the membranes. The bands were developed using ECL reagent (Tanon, Shanghai, China) and quantified using ImageJ software (Media Cybernetics, USA).

### RT-PCR

The total RNA was extracted with a TRIzol reagent (Beyotime, China). After quantitation on a NanoDrop-2000 spectrophotometer (Thermo Fisher, Massachusetts, USA), the RNA was reverse transcribed with equal volumes of RNase-free H_2_O (TaKaRa, Dalian, Japan). In a LightCycler® 480 (Roche Diagnostics International Ltd., Switzerland) with TB Green reagent, corresponding primers and RNase-free water were mixed for real-time PCR amplification. For normalization and quality control, GAPDH was used as a housekeeping gene. The sequences of primers applied are listed in Table 2. The relative mRNA expression levels were quantified using the 2^−ΔΔCt^ method after normalization.

**Table 2:** Primers used for RT-PCR.

GENE	Primer/probe	Sequence
TRPA1	Forward Reverse	CAAGAAGGAGAGGCTGGA AAAGTCCGGGTGGCTAA
MMP9	Forward Reverse	CAAAGACCTGAAAACCTCCAAC GACTGCTTCTCTCCCATCATC
NFATc1	Forward Reverse	GAGAATCGAGATCACCTCCTAC TTGCAGCTAGGAAGTACGTCTT
GAPDH	Forward Reverse	GGTTGTCTCCTGCGACTTCA TGGTCCAGGGTTTCTTACTCC

### Immunocytochemistry

In a 24-well plate, the cells were cultured on coverslips and treated differently. After fixation with paraformaldehyde (4 wt%, Biosharp, China) and permeabilization with 0.1% Triton X-100 (Beyotime, China), the specimens were blocked with QuickBlock Blocking Solution (Beyotime, China). The primary antibodies, NFAT2 (Abclonal, A24872) and TRPA1 (Abclonal, A8568), were then added for incubation, followed by incubation with a fluorescent secondary antibody (Alexa Fluor 488 or Alexa Fluor 647, Abcam). Subsequently, DAPI (Beyotime, China) was used to counterstain the cell nuclei. Lastly, images were captured under a luminescence microscope, and fluorescence intensity was assessed with ImageJ software.

### Bone resorption assay

RAW 264.7 cells were cultured on bone slices (Amizona Scientific LLC, Hangzhou, China) with a density of 3 × 10^4^ cells/well. The cells were then treated with RANKL to induce osteoclast formation. Images of osteoclasts were captured using an inverted microscope (Zeiss, Germany), and the absorption area was measured via ImageJ software (Media Cybernetics, USA).

### RNA sequencing

The cells were divided into two categories, each with three replicates. Total RNA was extracted using TRIzol reagent (Beyotime, China) three days after the intervention. After determining the concentration of RNA in each specimen with Qubit (Thermo Fisher, USA), the samples were delivered to GENEWIZ (Suzhou, China) for further analysis. FPKMs were calculated using Cufflinks, and read counts were obtained through HTSeq-count. The DESeq (2012) R package was used to analyze differential expression, with a threshold for significantly differential expression set as a *p* value <0.05 and fold change <0.5 or >2. Different groups of genes were analyzed using hierarchical cluster analysis for differentially expressed genes (DEGs). Additionally, gene ontology (GO) was performed for enrichment analysis of DEGs using hypergeometric distribution.

### Fluorescence detection of ER morphology

The cells were cultured with 2 μM ER-RFP (Thermo Fisher, USA) for 10 h on coverslips in a 24-well plate. Subsequently, ER morphology was observed using confocal fluorescence microscopy (Zeiss, Germany). Representative fields were then displayed to observe the condition of ER pyknosis.

### Transmission electron microscopy (TEM)

The samples were stored at 4 °C with a glutaraldehyde solution. After fixation, the supernatant above the sediment was removed, and the samples were washed with PBS. Afterwards, the specimens were soaked in a 1% osmic acid solution. After washing, the sample was dehydrated with a gradient ethanol concentration. Subsequently, the sample was moved to undiluted acetone. A permeated sample was embedded and heated overnight at 70 °C. Sections of 70‒90 nm were obtained by slicing the sample using an ultrathin microslicer (Leica Microsystems, Solms, Germany). The sections were stained with lead citrate solution and uranyl acetate in a 50% ethanol-saturated solution, then dried and examined under a TEM (Hitachi, Tokyo, Japan).

### Measurement of ROS levels

In 6-well plates, cells were planted with a density of 10^5^ cells/well and treated with corresponding interventions at appropriate concentrations. Following the collection and treatment with DCFH-DA, the cells were incubated at 37 °C. The samples were resuspended in DMEM without FBS after washing with PBS. Finally, the ROS levels were detected through flow cytometry (Beckman, California, USA).

In 24-well plates, cells were planted with different interventions and treated with DCFH-DA. The samples were incubated at 37 °C for 20 min. After washing, the samples were resuspended in DMEM without FBS and observed under a fluorescence microscope (Zeiss, Germany).

### Statistical analysis

GraphPad Prism software (version 9.0) was used to analyze the experimental outcomes. The values are represented as means ± standard deviations. Statistical analysis was performed using the student’s *t*-test between two groups. For analysis of the significant differences across multiple groups, the Kruskal‒Wallis one-way analysis of variance (ANOVA) was used. The difference at *p < 0.05 was considered significant, while the difference at **p < 0.01 was considered highly significant.

## Results

### TRPA1 was upregulated in the process of OP and osteoclast differentiation

**Fig. 1:**
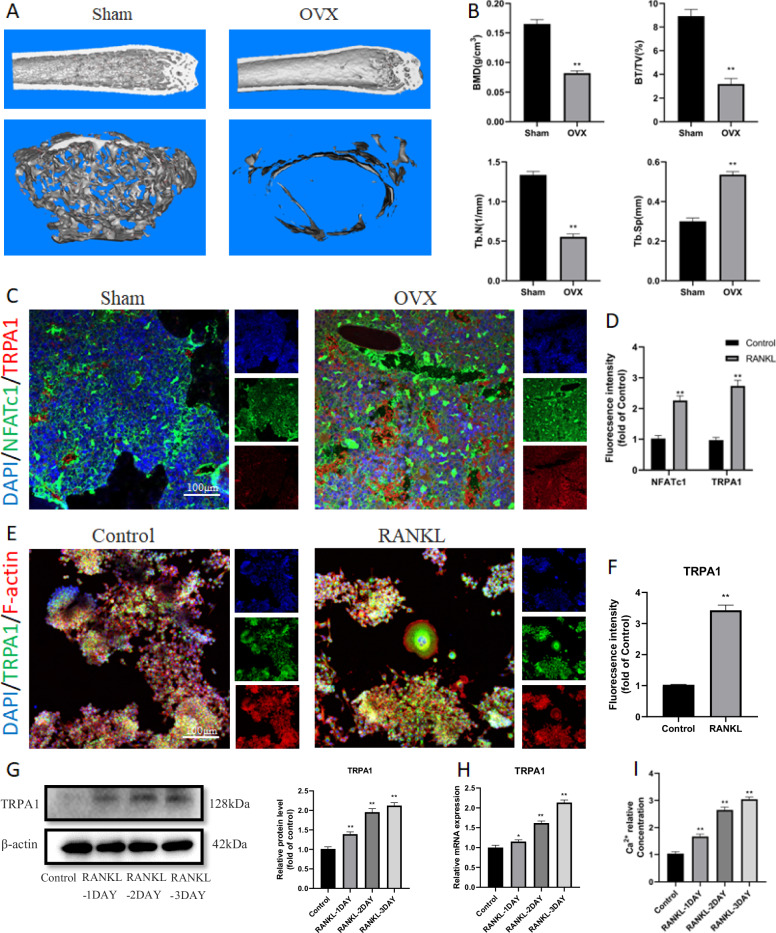
The establishment of the OVX-induced OP mouse model and TRPA1 was upregulated after surgery and RANKL intervention. **A** Representative pictures revealing that the femur osteopenia was induced by OVX. **B** Quantitative analysis of bone microstructure and cortical bone parameters. **C**, **D** Immunofluorescence staining and quantitation of NFATc1 and TRPA1 after surgery. *N* = 7 per group. **E**, **F** Immunofluorescence staining and quantitation of TRPA1 under RANKL intervention. Student’s *t*-test was applied to determine statistical significance. **G**, **H** Western blot and quantitation for TRPA1 expression in a dose-dependent manner for proteins of TRPA1 in a dose-dependent manner. **I** Intracellular calcium ion level in a dose-dependent manner. *N* = 3 per group. One-way ANOVA with Tukey’s multiple-comparison test was conducted to determine statistical significance (*p < 0.05, **p < 0.01).

First, the OP mouse models were established by bilateral oophorectomy. The micro-CT scanning revealed that the bone mass of the OVX group was significantly reduced compared to the Sham group 2 months after the operation (Fig. 1A, B). Immunofluorescence staining revealed that NFATc1 and TRPA1 were co-expressed in osteoclasts in the OVX group with a higher fluorescence intensity in comparison with the Sham group (Fig. 1C, D). RAW 264.7 cells were cultured in vitro and treated with RANKL. Correlated staining indicated that TRPA1 expression increased during osteoclastogenesis (Fig. 1E, F). Additionally, the western blot and RT-PCR analysis suggested that TRPA1 expression increased gradually during osteoclast formation (Fig. 1G, H). Meanwhile, the intracellular Ca^2+^ level exhibited a similar gradual increasing trend (Fig. 1I). These results suggested that TRPA1 was activated during the process of OP and osteoclast differentiation, indicating a potential connection between TRPA1 and OP.

### TRPA1 inhibition suppressed osteoclast differentiation and bone resorption

**Fig. 2:**
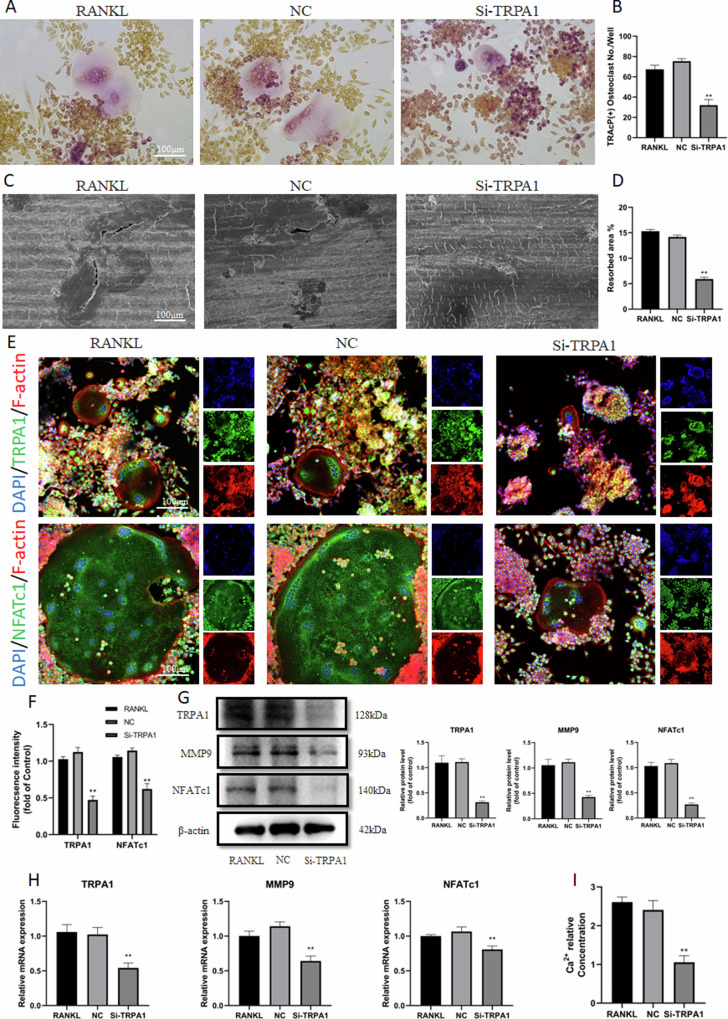
TRPA1 inhibition suppressed osteoclast differentiation and function. **A**, **B** Representative pictures of TRAcP staining and quantification of the TRAcP-positive cells (nuclei >3) under Si-TRPA1 intervention. **C**, **D** Representative pictures of bone resorption assay and quantification of resorbed area under Si-TRPA1 intervention. **E**, **F** Immunofluorescence staining and quantitation of TRPA1 and NFATc1 under Si-TRPA1 intervention. **G** Western blot and quantitation for proteins of TRPA1, MMP9, and NFATc1 under Si-TRPA1 intervention. **H** The relative mRNA expression of TRPA1, MMP9, and NFATc1 under Si-TRPA1 intervention. **I** Intracellular calcium ion level under Si-TRPA1 intervention. *N* = 3 per group. One-way ANOVA with Tukey’s multiple-comparison test was conducted to determine statistical significance (**p < 0.01).

A Si-RNA (Si-1091) significantly suppressed TRPA1 expression (Fig. S1A, B). CCK-8 suggested that 10 μM HC030031, an inhibitor of TRPA1, exhibited no impact on the viability of RAW 264.7 cells (Fig. S1D). TRAcP staining demonstrated that the quantity and area of positive cells were significantly reduced with TRPA1 inhibition (Fig. 2A, B and S2A, B). Additionally, bone resorption tests indicated that TRPA1 inhibition suppressed the resorbed area in comparison with the RANKL group (Fig. 2C, D). The immunofluorescence assay revealed the formation of osteoclasts after induction of RANKL, and TRPA1 inhibition significantly attenuated the fluorescence intensity of TRPA1 and NFATc1 (Fig. 2E, F and S2C, D). The Western blot and RT-PCR assay suggested that the expression of osteoclast-related genes, MMP9 and NFATc1, was significantly inhibited after TRPA1 silencing (Fig. 2G, H and S2E, F). A decrease in intracellular Ca^2+^ levels was observed after Si-TRPA1 intervention (Fig. 2I). TRPA1 inhibition declined the high intracellular Ca^2+^ level induced by RANKL (Fig. S2G). These results indicated that silencing TRPA1 could alleviate osteoclast activation and formation induced by RANKL.

### TRPA1 inhibition suppressed osteoclast differentiation and function by alleviating ER stress

**Fig. 3:**
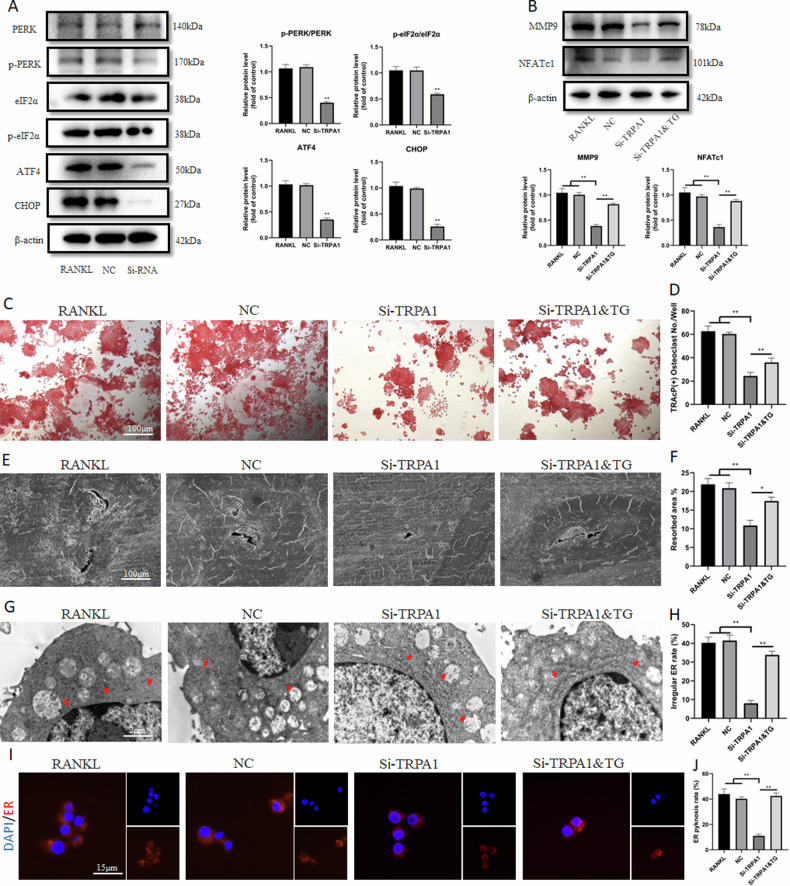
TRPA1 inhibition suppressed osteoclast differentiation and function by alleviating ER stress. **A** Western blot and quantitation for proteins of PERK/p-PERK, eIF2α/p- eIF2α, ATF4, and CHOP under Si-TRPA1 intervention. **B** Western blot and quantitation for proteins of TRPA1, MMP9, and NFATc1 under Si-TRPA1 and thapsigargin intervention. **C**, **D** Representative pictures of TRAcP staining and quantification of the TRAcP-positive cells (nuclei >3) under Si-TRPA1 and thapsigargin intervention. **E**, **F** Representative pictures of bone resorption assay and quantification of resorbed area under Si-TRPA1 and thapsigargin intervention. **G**, **H** Representative images and quantification of irregular ER intervened by Si-TRPA1 and thapsigargin under TEM. Red arrows represented ER. **I**, **J** Immunofluorescence staining of calnexin‐ER marker and quantification of ER pyknosis under Si-TRPA1 and thapsigargin intervention. *N* = 3 per group. One-way ANOVA with Tukey’s multiple-comparison test was conducted to determine statistical significance (*p < 0.05, **p < 0.01).

Previous research has revealed that the inhibition of ER stress-related pathways, such as PERK and IRE1α/XBP1, could significantly hinder the formation and bone resorption of osteoclasts [10, 11]. Additionally, other studies have reported that TRPA1 could activate ER stress to induce apoptosis, mitochondrial morphology, and dysfunction [16, 17]. Our findings suggested that the expression of the PERK/eIF2α/ATF4/CHOP pathway gradually increased with prolonged RANKL intervention time (Fig. S1C). We were interested in exploring the connection between TRPA1 and ER stress in osteoclasts. Figure 3A displays that the expression of the PERK pathway was down-regulated with Si-TRPA1 intervention.

For further verification, one of the agonists of ER stress, thapsigargin, was applied. The CCK-8 indicated that 0.1 nM thapsigargin exhibited no influence on the viability of RAW 264.7 cells (Fig. S1E). A Western blot assay revealed that the protein expression of MMP9 and NFATc1 was significantly upregulated after ER stress induction (Fig. 3B). Compared with the Si-TRPA1 group, the quantity and area of TRAcP-positive cells increased with thapsigargin intervention (Fig. 3C, D). To investigate whether ER stress influences bone resorption, a hydroxyapatite-coated plate was used to test the mature osteoclast’s ability to resorb bone matrix. Si-TRPA1 treatment inhibited resorbing activity, but the absorption area was significantly increased after ER stress induction (Fig. 3E, F). These results suggested that thapsigargin intervention significantly promoted osteoclastic differentiation and function. TEM was used to identify intracellular organelles, and it was found that the morphology of ER became swollen and irregular with RANKL induction. TRPA1 inhibition made it smooth and regular, while thapsigargin intervention aggravated this morphological irregularity (Fig. 3G, H). Figure 3I, J depict that thapsigargin intervention reversed the reduced ER granularity through TRPA1 inhibition. However, the intracellular Ca^2+^ levels of the thapsigargin intervention group had no clear change compared to that of the Si-TRPA1 group (Fig. S3A). These results indicated that TRPA1 silencing could suppress osteoclast differentiation and function by alleviating ER stress.

### TRPA1 inhibition suppressed osteoclast differentiation by alleviating ER stress mediated by SRXN1

**Fig. 4:**
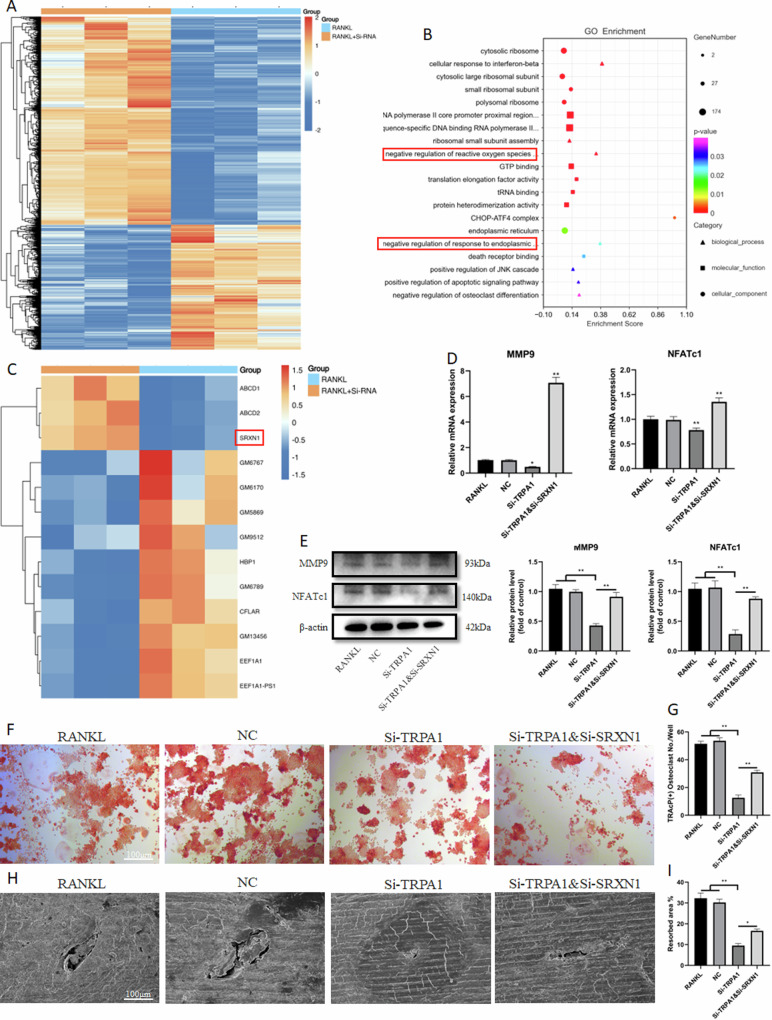
TRPA1 inhibition suppressed osteoclast differentiation via alleviating ER stress mediated by SRXN1. **A** The differentially expressed mRNAs in RAW 264.7 cells in response to Si-TRPA1 are illustrated as a heatmap. **B** GO enrichment of differential genes. **C** Heatmap of differential genes in negative regulation of reactive oxygen species biosynthetic process. **D** The relative mRNA expression of MMP9 and NFATc1 under Si-TRPA1 and Si-SRXN1 intervention. **E** Western blot and quantitation for proteins of MMP9 and NFATc1 under Si-TRPA1 and Si-SRXN1 intervention. **F**, **G** Representative images of TRAcP staining and quantification of the TRAcP-positive multinucleated cells (nuclei >3) under Si-TRPA1 and Si-SRXN1 intervention. **H**, **I** Representative images of bone resorption assay and quantification of the resorbed area under Si-TRPA1 and Si-SRXN1 intervention. *N* = 3 per group. One-way ANOVA with Tukey’s multiple-comparison test was conducted to determine statistical significance (*p < 0.05, **p < 0.01).

We conducted RNA sequencing on RAW 264.7 cells induced with RANKL and treated with or without Si-TRPA1 to better understand how TRPA1 inhibits osteoclast function. In comparison with RANKL-only cells, 2901 DEGs were identified in cells treated with RANKL and Si-TRPA1 (fold change >1.5 and FDR <0.05) (Fig. 4A). To confirm gene function, GO terms were further analyzed. Notably, negative regulation of the ROS biosynthetic process and ER stress are enriched in biological processes (Fig. 4B). Flow cytometry and ROS assays were employed to verify these findings, revealing that ROS production was significantly reduced after TRPA1 inhibition. Compared with the RANKL group, the levels of ROS decreased in the Si-TRPA1 group (Fig. S3C‒F). Additionally, the ER was marked with RFP and observed as granular under laser confocal microscopy, indicating morphological changes in the ER during ER stress. Fig. S3E, F display that ER presented obvious granular morphology in the RANKL group, while this change was reduced after TRPA1 inhibition.

After analysis and visualization of differential genes in the negative regulation of the ROS biosynthetic process during TRPA1 silencing, we observed that SRXN1, an antioxidant gene, was significantly down-regulated (Fig. 4C). Further PCR results confirmed this alteration, and Si-SRXN1 was applied for subsequent verification (Fig. S1F, G). The mRNA and protein expression of MMP9 and NFATc1 decreased with TRPA1 inhibition, while they increased after both TRPA1 and SRXN1 silencing (Fig. 4D, E). TRAcP staining revealed that SRXN1 silencing reversed the reduced quantity and area of osteoclasts by TRPA1 inhibition (Fig. 4F, G). The bone resorption test also suggested that SRXN1 silencing promoted the resorbed area in comparison with the TRPA1 inhibition group (Fig. 4H, I). These results indicated that SRXN1 influenced osteoclast differentiation and function during TRPA1 inhibition.

**Fig. 5:**
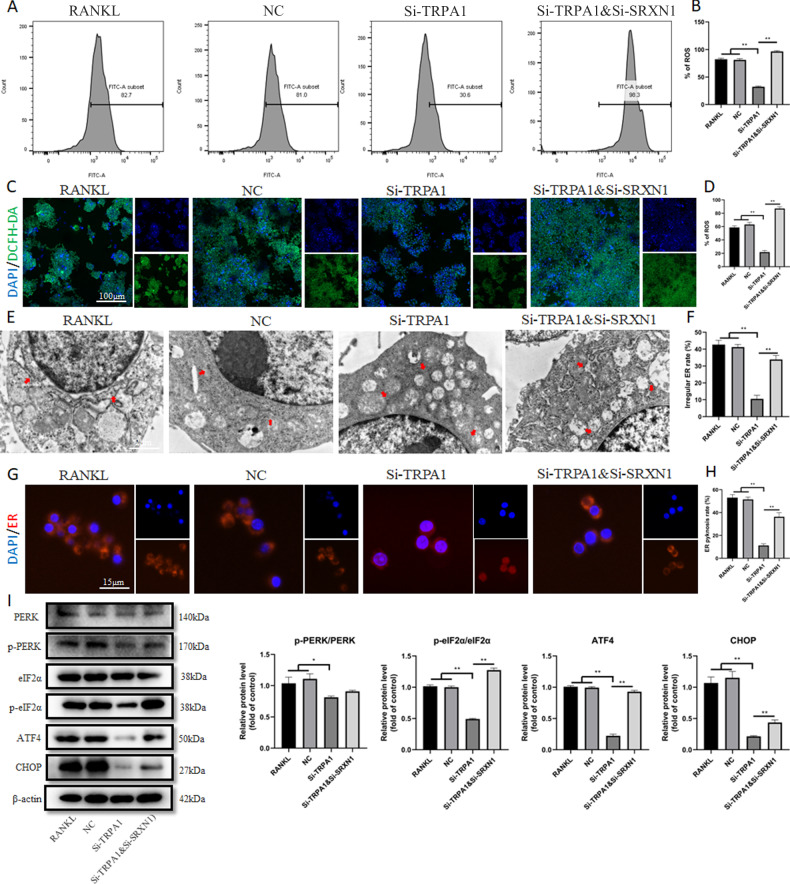
TRPA1 inhibition suppressed osteoclast differentiation via alleviating ER stress mediated by SRXN1. **A**, **B** Flow cytometric analysis and quantitation of ROS-positive cells under Si-TRPA1 and Si-SRXN1 intervention. **C**, **D** Representative images of and quantitation of ROS-positive cells under Si-TRPA1 and Si-SRXN1 intervention. **E**, **F** Representative images and quantification of irregular ER intervened by Si-TRPA1 and Si-SRXN1 and thapsigargin under TEM. Red arrows represented ER. **G**, **H** Immunofluorescence staining of calnexin‐ER marker and quantification of ER pyknosis under Si-TRPA1 and Si-SRXN1 intervention. **I** Western blot and quantitation for proteins of PERK/p-PERK, eIF2α/p- eIF2α, ATF4, and CHOP under Si-TRPA1 and Si-SRXN1 intervention. *N* = 3 per group. One-way ANOVA with Tukey’s multiple-comparison test was conducted to determine statistical significance (*p < 0.05, **p < 0.01).

Further research was conducted to observe the antioxidant function of SRXN1 and its connection with ER stress. The flow cytometry assay demonstrated that TRPA1 inhibition substantially reduced the ROS level, which had increased with SRXN1 silencing (Fig. 5A, B). In comparison with the TRPA1 inhibition group, the levels of ROS also increased in the TRPA1 and SRXN1 inhibition groups (Fig. 5C, D). Figure 5E, F demonstrate that TRPA1 inhibition relieved the irregular morphology of ER induced by RANKL, but SRXN1 silencing reversed this change. Fluorescence staining revealed that the ER granules were reduced with TRPA1 inhibition, while they reappeared after SRXN1 silencing (Fig. 5G, H). We also observed that the protein expression of the PERK pathway exhibited similar variations to ROS synthesis, indicating that the production of ROS regulated by SRXN1 could affect ER stress (Fig. 5I). But the intracellular Ca^2+^ levels of the Si-SRXN1 group also had no significant change in comparison with that of the Si-TRPA1 group (Fig. S3B). These results revealed that ER stress mediated by ROS-influenced osteoclast differentiation and function during TRPA1 inhibition.

### TRPA1 inhibition prevents OVX-induced bone loss in vivo

**Fig. 6:**
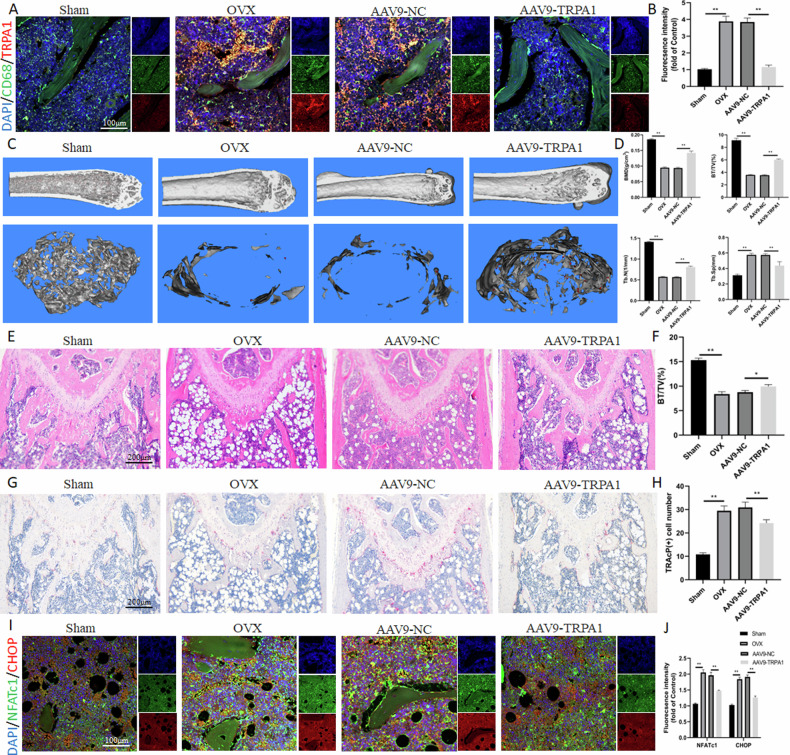
TRPA1 inhibition prevents OVX-induced bone loss in vivo. **A**, **B** Immunofluorescence staining and quantitation of TRPA1 under AAV9-TRPA1 intervention. **C** Representative pictures revealed that the femur bone loss was alleviated under AAV9-TRPA1 intervention. **D** Quantitative analysis of parameters regarding bone microstructure and cortical bone. **E**, **F** H&E staining and quantitative analysis of histomorphometric bone parameters of BV/TV (%). **G**, **H** TRAcP staining and quantitative analysis of TRAcP+ osteoclast number in the bone sections. **I**, **J** Immunofluorescence staining and quantitation of NFATc1 and CHOP under AAV9-TRPA1 intervention. *N* = 7 per group. One-way ANOVA with Tukey’s multiple-comparison test was conducted to determine statistical significance (**p < 0.01).

To evaluate the function of TRPA1 in OVX-induced osteopenia, AAV9 was used to knock down TRPA1 expression in the bone marrow-derived macrophages of OVX mice. Immunofluorescent staining indicated that the expression of TRPA1 increased after OVX induction; however, it was successfully suppressed by AAV9-TRPA1 (Fig. 6A, B). In OVX mice, AAV9-TRPA1 protected against bone loss in the cancellous bone of the distal femur. Three-dimensional reconstructions and volume measurements from micro-CT scans revealed that BMD decreased in the OVX group, but AAV9-TRPA1 treatment reversed this decline, demonstrating its validity for OP. Moreover, additional factors such as Tb. N, BV, BV/TV, and Tb. Sp indicated that AAV9-TRPA1 decreased osteopenia caused by estrogen deficiency. (Fig. 6C, D). Hematoxylin and eosin staining revealed that BV/TV (%) was higher in the treated groups with AAV9-TRPA1 compared to the OVX group (Fig. 6E, F). Additionally, bone trabeculae osteoclast numbers were assessed to determine how TRPA1 deficiency affects bone resorption in vivo. TRAcP staining indicated that the count of osteoclasts was reduced in the AAV9-TRPA1 group compared with the OVX group, suggesting that TRPA1 inhibition could alleviate OP by suppressing osteoclast formation (Fig. 6G, H). Compared with the Sham group, the expression of NFATc1 and CHOP increased in the OVX group while decreasing in the AAV9-TRPA1 group (Fig. 6I, J). This result further confirmed that TRPA1 inhibition prevented bone loss in vivo by suppressing ER stress.

## Discussion

OP in elderly individuals is characterized by low bone mass, weak muscle power, and a high fracture risk [19]. Currently, 200 million people globally are affected by OP, with approximately 8.9 million patients suffering from fractures each year [20, 21]. It is believed that aging and estrogen deficiency play the most crucial roles in the pathogenesis of postmenopausal OP [22]. Therefore, understanding the molecular mechanism of OP and identifying more efficient therapeutic targets is essential for its treatment.

The homeostasis of the skeleton is crucial for maintaining long-term bone health, as bone biology is regulated by various physiological factors. Osteoclast bone resorption and osteoblast bone formation are responsible for bone metabolism. Promoting osteogenic differentiation or inhibiting osteoclast differentiation can alleviate OP [23, 24]. Abnormal osteoclast differentiation is the primary pathological cause of OP, making osteoclasts the primary target for OP prevention [25]. Consequently, inhibiting progressive osteoclast hyperactivity could be a valuable therapeutic approach.

TRPA1, a member of the superfamily of TRP cation channels, exerts anti-oxidative and anti-ER stress effects across various diseases [26–28]. It was observed that TRPA1 is activated in osteoclasts in the OVX mice, and its expression is upregulated during osteoclast differentiation. This suggests a potential connection between TRPA1 and osteoclasts. Further experiments revealed that TRPA1 inhibition significantly suppressed osteoclast differentiation and function and alleviated bone loss after surgery. These results indicated that TRPA1 could be an effective therapeutic target for OP; however, related molecular mechanisms remain unknown. Additionally, the activation of TRPA1 results in PERK-mediated ER stress initiation in human bronchial epithelial cells, indicating increased expression of pro-apoptotic markers such as CHOP and ATF3 [29]. Viisanen et al. reported that TRPA1-mediated pain hypersensitivity is induced by both diabetic and non-diabetic ER stress [30]. As a result, it is essential to further explore the role of ER stress in osteoclast differentiation and whether TRPA1 could influence ER stress during osteoclastogenesis.

Several diseases are associated with ER stress, including inflammatory responses, metabolic disorders, neurodegenerative disorders, and immune deficiencies [31]. The UPR is an evolutionary conserved cellular mechanism that deals with ER homeostasis imbalance and regulates cell function. Studies have indicated that genes associated with ER stress are activated during osteoclast formation, suggesting a connection between osteoclastogenesis and ER stress [32]. The PERK and IRE1α signaling pathways aggravated osteoclast formation via activating the MAPK, NF-κB signaling, or releasing pro-inflammatory factors [33]. Besides, osteoclast differentiation is promoted by the RANKL/receptor activator of NF-κB through binding XBP1 to NFATc1 [34]. Current evidence also revealed that the UPR signaling pathway could indirectly enhance osteoclastogenesis through increased secretion of RANKL from osteoblasts [35]. Our findings revealed the activation of the PERK pathway during osteoclast formation, implying a close association between ER stress and osteoclastogenesis. Additionally, subsequent outcomes demonstrated that the suppression of URP could inhibit osteoclast formation and function. Wei et al. found that the knockout of PERK significantly reduced the expression of osteoclast-related genes, such as TRAP and cathepsin K [36]. Hamamura et al. reported that the inhibition of eIF2α dephosphatase reduced NFATc1 expression, thus alleviating osteoclast formation [37]. Therefore, further research is required to explore the underlying mechanism of ER stress influencing osteoclastogenesis.

Subsequently, we investigated the influence of TRPA1 on ER stress during osteoclast differentiation and found that silencing TRPA1 inhibited the activation of the PERK pathway during osteoclast differentiation. In vivo, the fluorescence intensity of CHOP also decreased after AAV9-TRPA1 intervention, mainly co-expressed with NFATc1 in osteoclasts. Wang et al. discovered that TRPA1 inhibitors could alleviate inflammation, oxidative stress, mitochondrial dysfunction, ER stress, and apoptosis in cardiomyocytes [16]. We hypothesized that TRPA1 inhibition could influence ER stress and osteoclastogenesis. To investigate the role of TRPA1 in ER stress and osteoclastogenesis, we used thapsigargin, a traditional agonist of ER stress. Guo et al. reported that the formation of osteoclasts is facilitated by ER stress at an appropriate level [10]. Lee et al. demonstrated that thapsigargin promotes the NF-κB pathway by activating PERK and BiP, and then increases the expression of NFATc1 and TRAP and aggravates osteoclast formation and bone resorption [38]. In addition, ER stress induced by thapsigargin activates NFATc1 and c-fos via the BiP-PERK-eIF2α signaling pathway to enhance osteoclastogenesis [39]. Our results revealed that thapsigargin intervention successfully promoted ER stress in osteoclasts and reversed the suppression of osteoclast differentiation and function by TRPA1 inhibition. These results demonstrated that TRPA1 inhibition could suppress osteoclastogenesis by alleviating ER stress.

Our RNA-seq analysis revealed that TRPA1 inhibition caused the activation of SRXN1, which was confirmed by RT-PCR. Subsequent experiments confirmed that silencing SRXN1 partially weakened the suppression of osteoclast differentiation and function influenced by TRPA1 inhibition. SRXN1 was known as an antioxidant molecule involved in the maintenance of oxidative homeostasis, and we were curious about how SRXN1 affected osteoclastogenesis [40]. Recent research indicated that inflammatory injury and oxidative stress caused by high glucose are prevented by SRXN1 in the retinal ganglion cells [41]. Another study revealed that ROS and ER stress are attenuated by SRXN1 in acute pancreatitis [42]. Our results suggested that SRXN1 silencing significantly increased the generation of ROS. Besides, the expression of the PERK pathway was upregulated, and ER stress was promoted with SRXN1 silencing.

Research suggested the accumulation of unfolded proteins in the ER could lead to mitochondrial ROS production [43]. Wang et al. revealed that ER stress promotes intracellular ROS in SH-SY5Y cells to regulate parthanatos induced by oxygen-glucose deprivation [44]. However, there is growing evidence that ROS could activate ER stress. For instance, resveratrol and arsenic trioxide induce apoptosis in human lung adenocarcinoma cells through ROS-mediated ER stress [45]. Smoking induces apoptosis in human bronchial epithelial cells through CHOP and ROS-dependent ER stress [46]. We found that the silencing of the antioxidant protein SRXN1 increased ROS production, enhanced PERK expression, and promoted ER stress. It is speculated that TRPA1 inhibition suppresses osteoclast differentiation by alleviating ER stress mediated by ROS. Although oxidative and ER stress are closely associated, molecular events involving ROS and ER stress are still not fully understood.

The present study indicated that the inhibition of TRPA1 prevented osteoclast differentiation by suppressing ER stress. In vivo, the targeted inhibition of TRPA1 alleviated bone loss in OVX mice. Mechanistically, TRPA1 inhibition suppressed ROS-induced ER stress, thereby reducing osteoclast differentiation.

However, there are some limitations to the study. The murine leukemia macrophage cell RAW 264.7 is a type of immortal cell that cannot replace primary cells to simulate physiology. Additionally, TRPA1 is a calcium channel responsible for Ca^2+^ influx. Further research is required to explore how TRPA1 influences Ca^2+^ influx to promote ROS production and subsequent ER stress.

## Conclusion

Our findings indicated that TRPA1 inhibition relieved OVX-induced bone loss by suppressing osteoclast formation. In terms of mechanism, in vitro results revealed that TRPA1 inhibition alleviated ROS and ER stress. Moreover, blocking the expression of the antioxidant gene SRXN1 aggravated ER stress induced by RANKL. Moreover, TRPA1 inhibition prevented osteopenia induced by OVX in vivo. Our study revealed the regulatory role of TRPA1 on ER stress and osteoclastogenesis, providing new perspectives for understanding the mechanism of osteoclast differentiation.

### Supplementary information

Supplementary Information

## Data Availability

The original contributions presented in the study are included in the article material, further inquiries can be directed to the corresponding author/s. All methods were performed in accordance with the relevant guidelines and regulations.
