# Supplementary material for: TRPA1 aggravates osteoclastogenesis and osteoporosis through activating endoplasmic reticulum stress mediated by SRXN1
Source: Cell Death Dis. 2024 Aug 27;15(8):624. doi: 10.1038/s41419-024-07018-5 (PMC11349872; doi:10.1038/s41419-024-07018-5)

Supplementary Information

Materials and methods

Cytotoxicity assay

An MTS assay kit was used to evaluate the cytotoxicity of HC030031 and thapsigargin before the in vitro experiment. In 96-well plates, the RAW 264.7 cells (5 × 10^3/well) were cultured with appropriate DMEM. HC030031 and thapsigargin were added to each well and incubated for 24 h at different concentrations. Subsequently, the cells were incubated for 3 h with MTS solution (100 μL/well). A microplate reader (BioTek, USA) was employed to measure the optical density at 450 nm to evaluate the effects of HC030031 and thapsigargin on cell proliferation.

Figure. S1

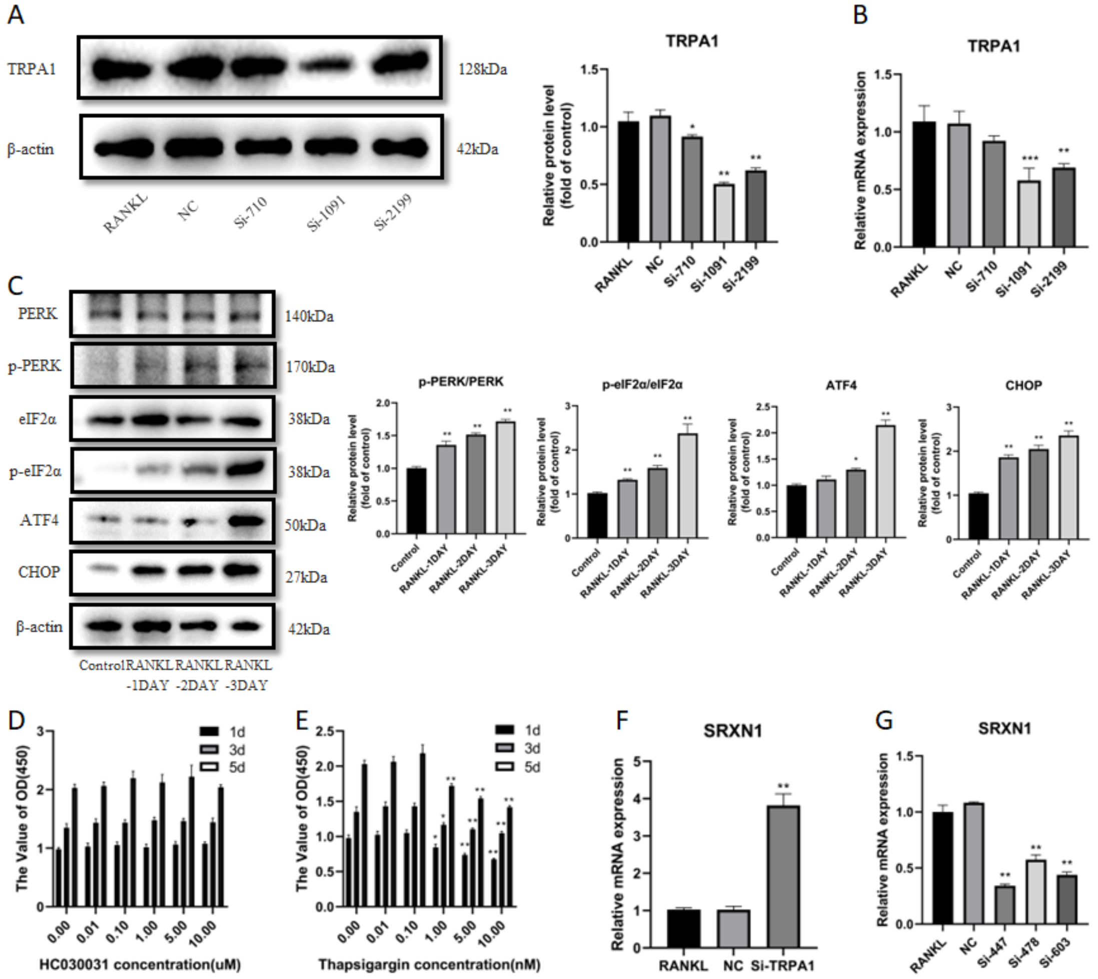
A
TRPA1
128kDa
β-actin
42kDa
RANKL
NC
Si-710
Si-1091
Si-2199
TRPA1
Relative protein level (fold of control)
B
TRPA1
Relative mRNA expression
C
PERK
140kDa
p-PERK
170kDa
eIF2α
38kDa
p-eIF2α
38kDa
ATF4
50kDa
CHOP
27kDa
β-actin
42kDa
Control
RANKL -1DAY
RANKL -2DAY
RANKL -3DAY
p-PERK/PERK
p-eIF2α/eIF2α
ATF4
CHOP
D
The Value of OD(450)
1d
3d
5d
HC030031 concentration(uM)
E
Thapsigargin concentration(nM)
F
SRXN1
Relative mRNA expression
RANKL
NC
Si-TRPA1
G
SRXN1
Si-447
Si-478
Si-603

Figure. S2

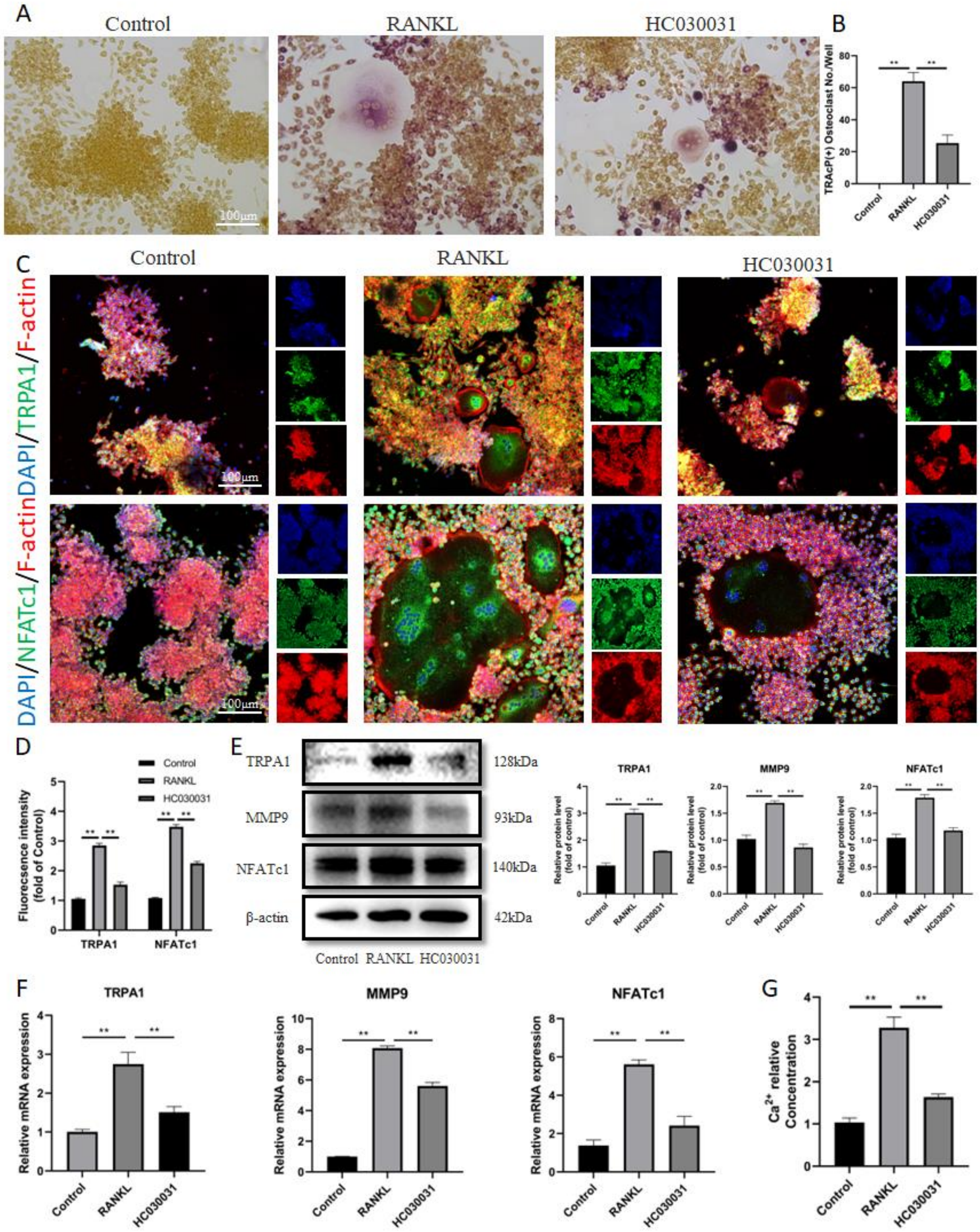
A
Control
RANKL
HC030031
100μm
B
TRAcP(+) Osteoclast No./Well
Control
RANKL
HC030031
C
DAPI/TRPA1/F-actin
DAPI/NFATc1/F-actin
Control
RANKL
HC030031
100μm
D
Fluorescence intensity (fold of Control)
Control
RANKL
HC030031
TRPA1
NFATc1
E
TRPA1
MMP9
NFATc1
β-actin
128kDa
93kDa
140kDa
42kDa
Control RANKL HC030031
Relative protein level (fold of control)
F
TRPA1
MMP9
NFATc1
Relative mRNA expression
Control
RANKL
HC030031
G
Ca2+ relative Concentration
Control
RANKL
HC030031

Figure. S3

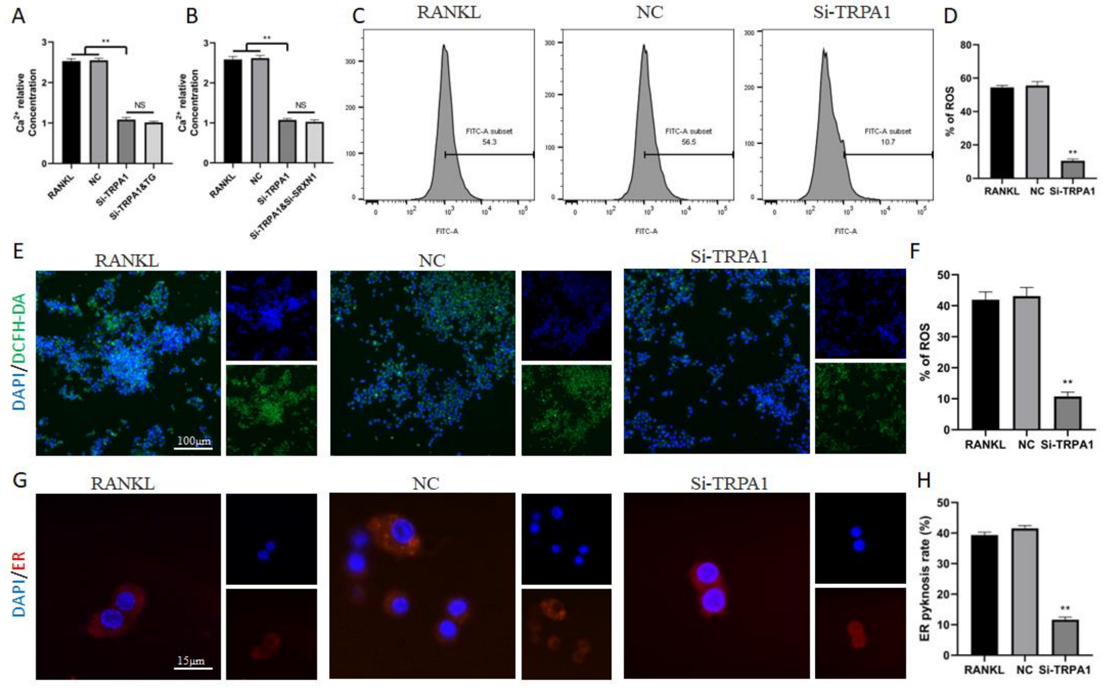
A
B
C
D
E
F
G
H
Ca²⁺ relative Concentration
RANKL
NC
Si-TRPA1
Si-TRPA1&TG
Si-TRPA1&Si-SRXN1
**
NS
FITC-A subset
54.3
56.5
10.7
FITC-A
% of ROS
DAPI/DCFH-DA
100μm
DAPI/ER
15μm
ER pyknosis rate (%)

Figure legends

Fig. S1 Verification of inhibition effect of TRPA1 and cell viability assay. **(A)** Western blot and quantitation for proteins of TRPA1 under Si-TRPA1 intervention. **(B)** The relative mRNA expression of TRPA1 under Si-TRPA1 intervention. **(C)**Western blot and quantitation for proteins of PERK/p-PERK, eIF2α/p- eIF2α, ATF4 and CHOP under RANKL intervention. **(D-E)** CCK8 assay on different concentration and different stimulation time of HC030031 and thapsigargin in RAW 264.7 cells. **(F)** The relative mRNA expression of SRXN1 under Si-TRPA1 intervention. **(G)** The relative mRNA expression of SRXN1 under Si-SRXN1 intervention. N=3 per group. One-way ANOVA with Tukey's multiple-comparison test was conducted to determine statistical significance (*$p < 0.05$, **$p < 0.01$).

Fig. S2 TRPA1 inhibition suppressed osteoclast differentiation. **(A-B)** Representative images of TRAcP staining and quantification of the TRAcP-positive multinucleated cells (nuclei > 3) under HC030031 intervention. **(C-D)** Immunofluorescence staining and quantitation of TRPA1 and NFATc1 under HC030031 intervention. **(E)** Western blot and quantitation for proteins of TRPA1, MMP9 and NFATc1 under HC030031 intervention. **(F)** The relative mRNA expression of TRPA1, MMP9 and NFATc1 under HC030031 intervention. **(G)** Intracellular calcium ion level under HC030031 intervention. N=3 per group. One-way ANOVA with Tukey's multiple-comparison test was conducted to determine statistical significance (**$p < 0.01$).

Fig. S3 ER stress was activated during osteoclastic differentiation and TRPA1 inhibition reduced ROS production. **(A-B)** Intracellular calcium ion level under thapsigargin and Si-SRXN1 intervention. **(C-D)** Flow cytometric analysis and quantitation of ROS positive cells under Si-TRPA1 intervention. **(E-F)** Representative images of and quantitation of ROS positive cells under Si-TRPA1 intervention. **(G-H)** Immunofluorescence staining of calnexin-ER marker and quantification of ER pyknosis under Si-TRPA1 intervention. N=3 per group. One-way ANOVA with Tukey's

multiple-comparison test was conducted to determine statistical significance (*$p < 0.05$, **$p < 0.01$).

Original western blots

Blots from Fig1. G

TRPA1

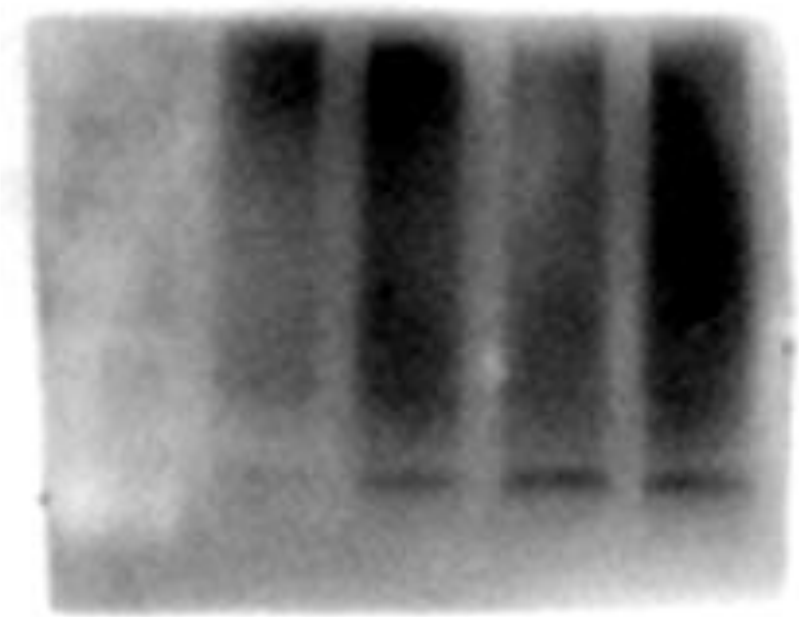

β-actin

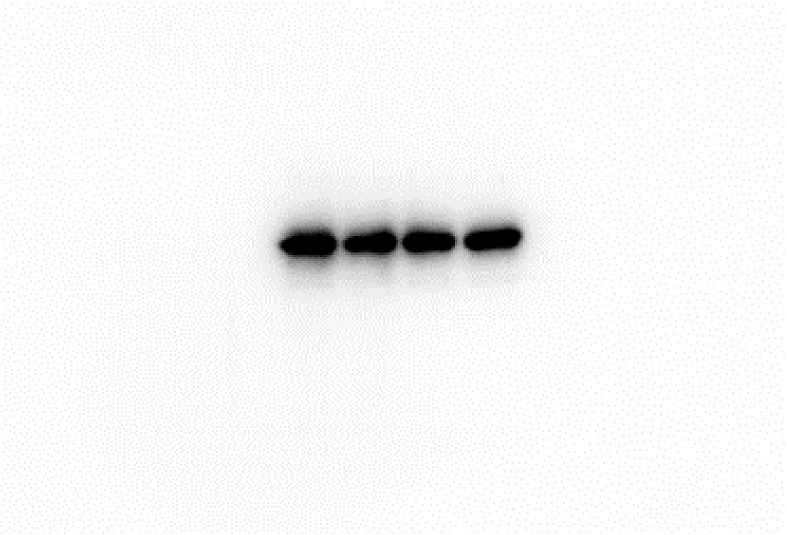

Blots from Fig2. G

TRPA1

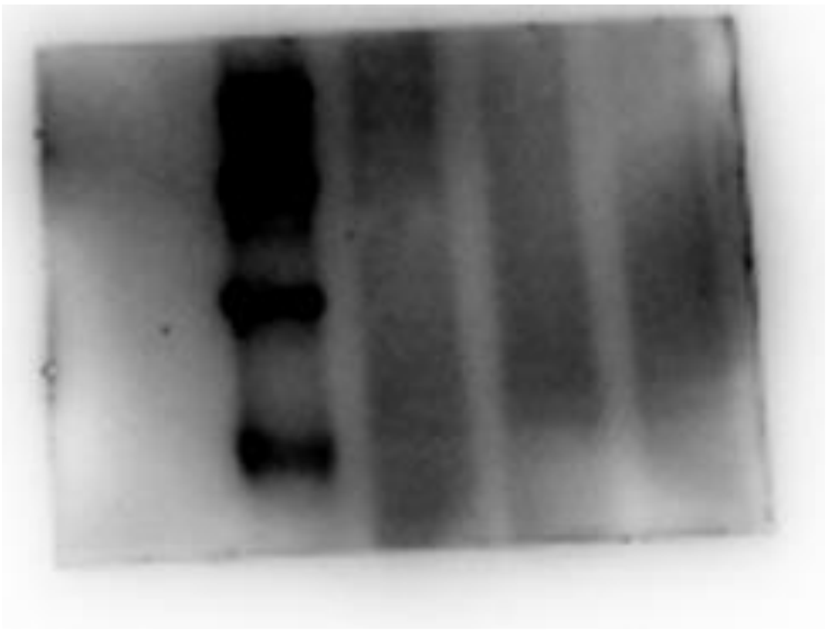

MMP9

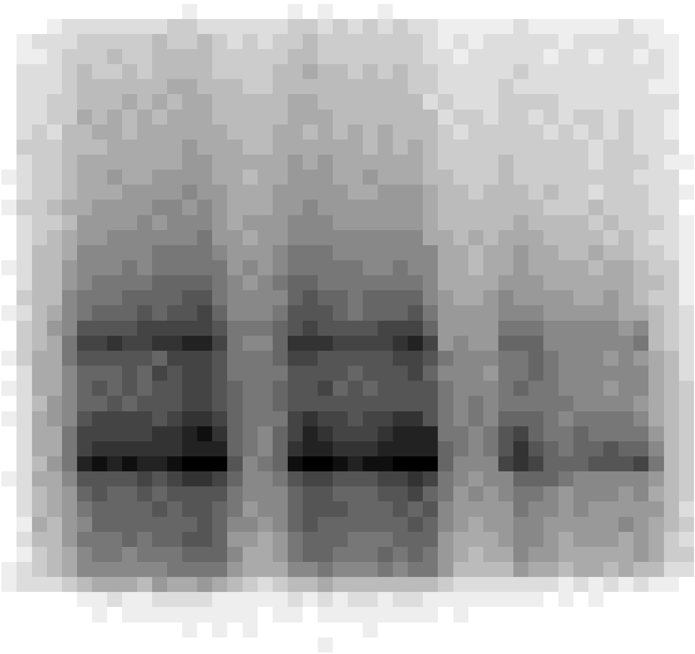

NFATc1

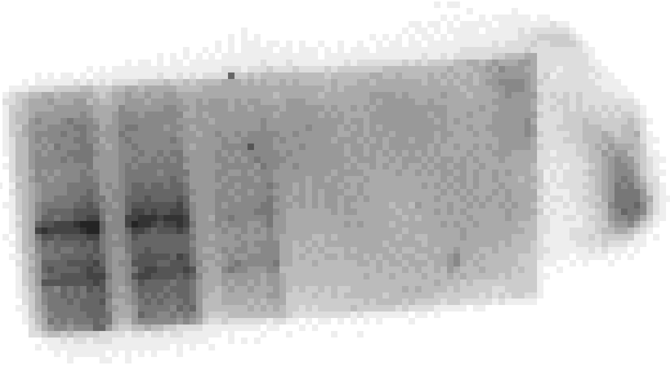

β-actin

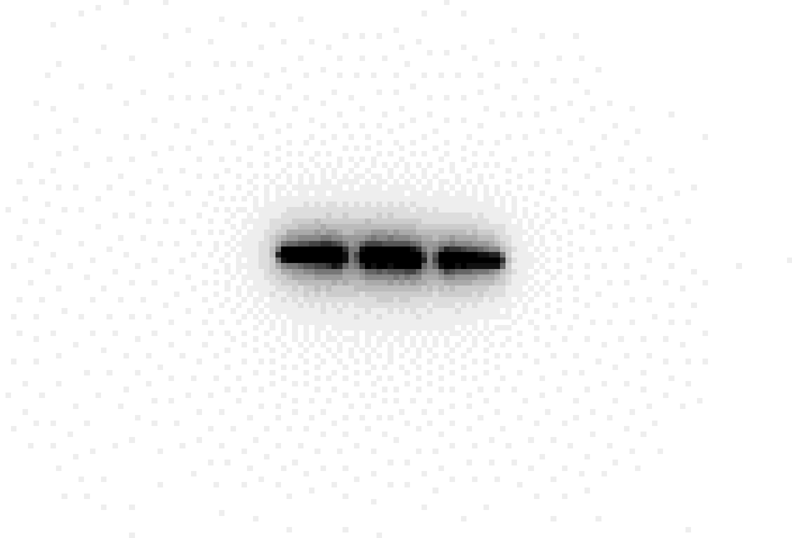

Blots from Fig3. A

PERK

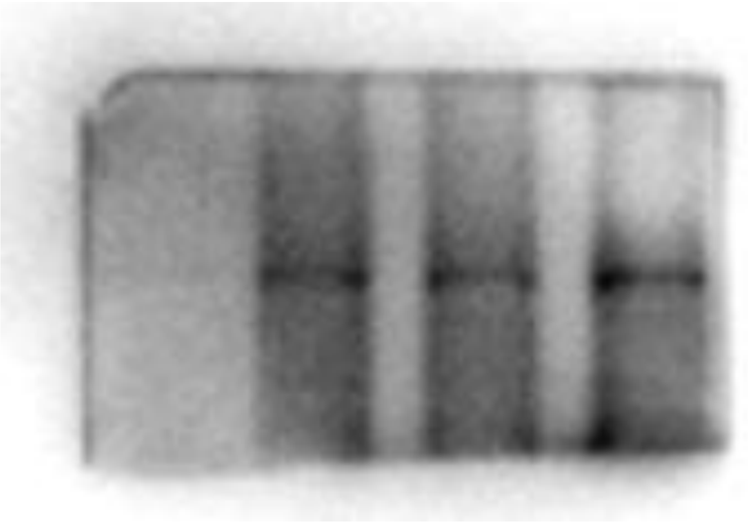

p-PERK

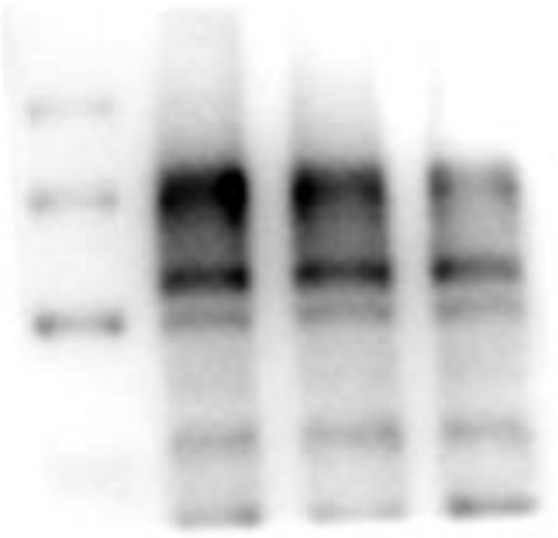

eIf2α

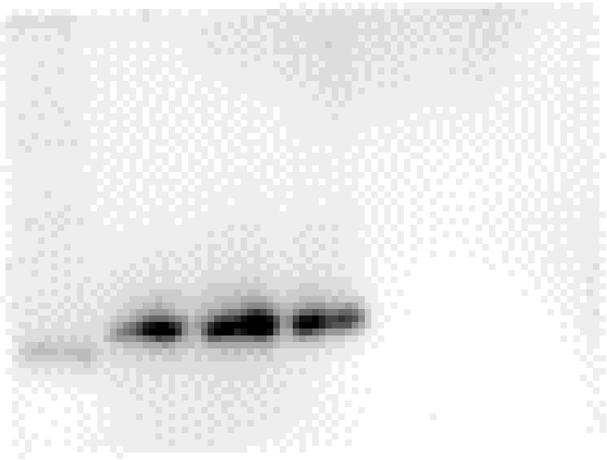

p-eIf2α

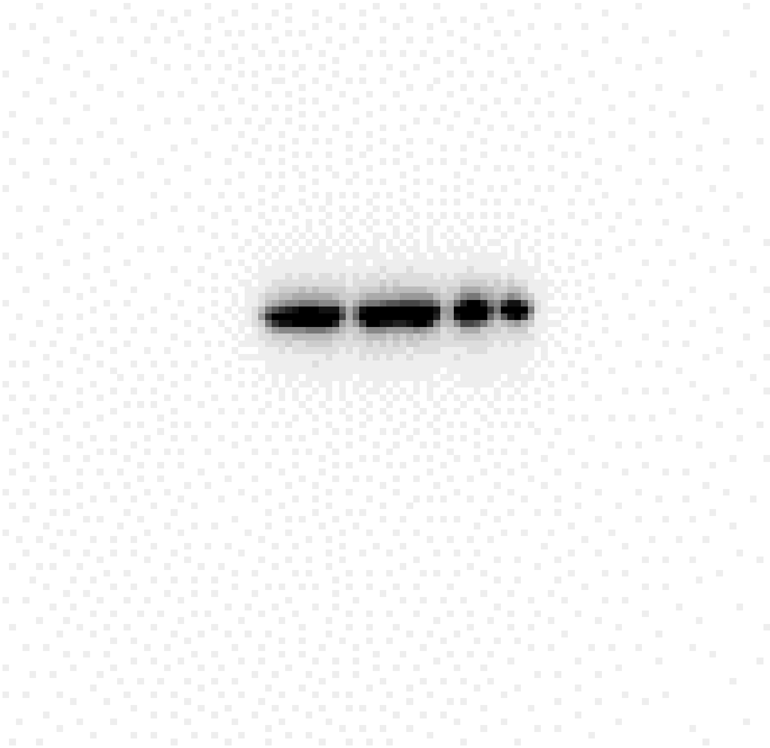

ATF4

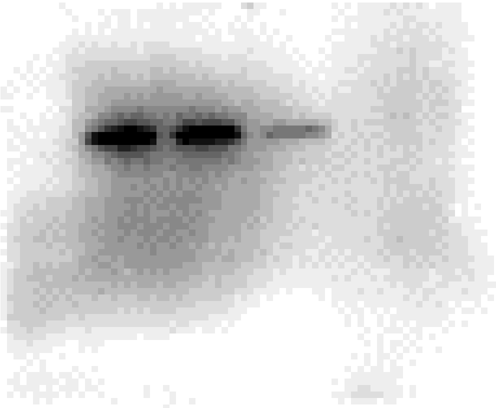

CHOP

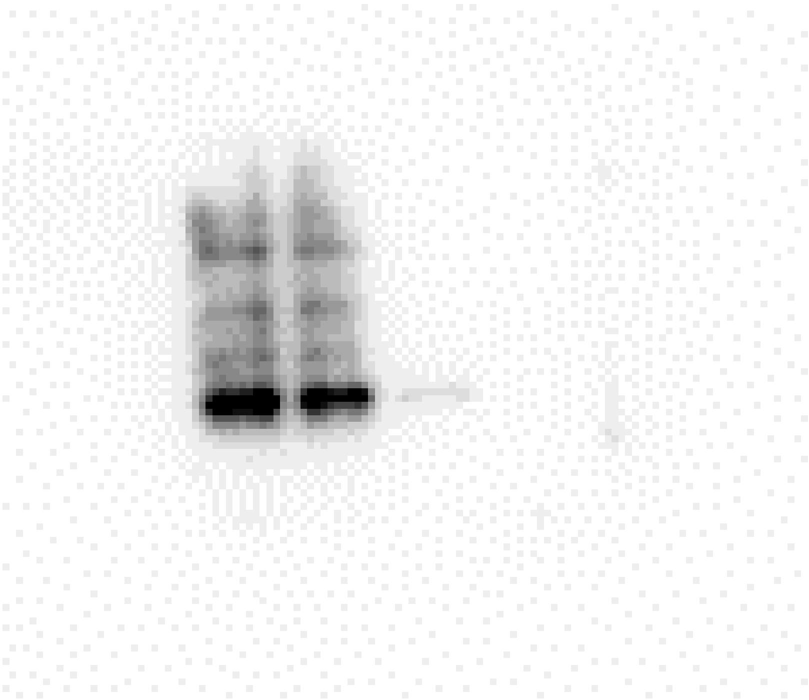

β-actin

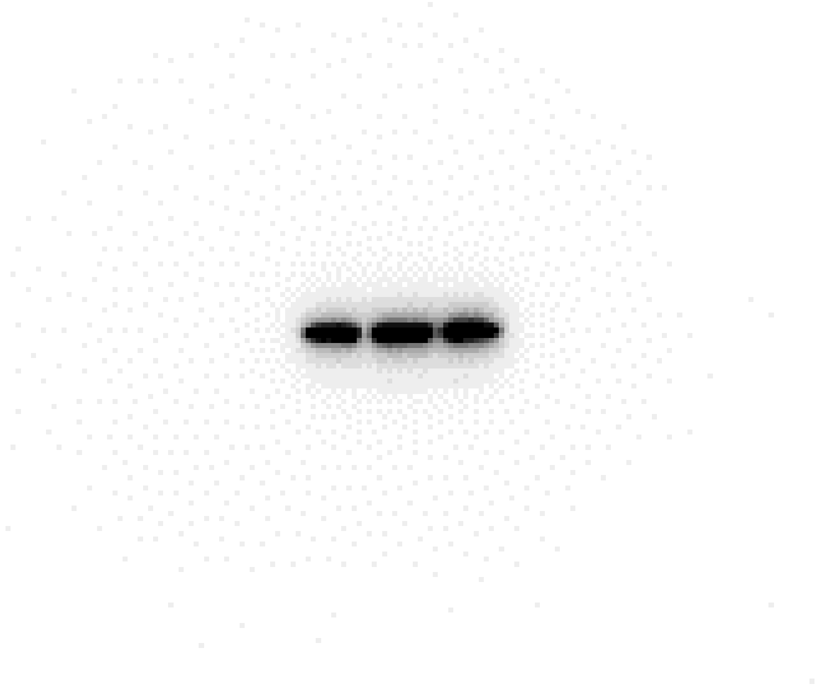

Blots from Fig3. B

MMP9

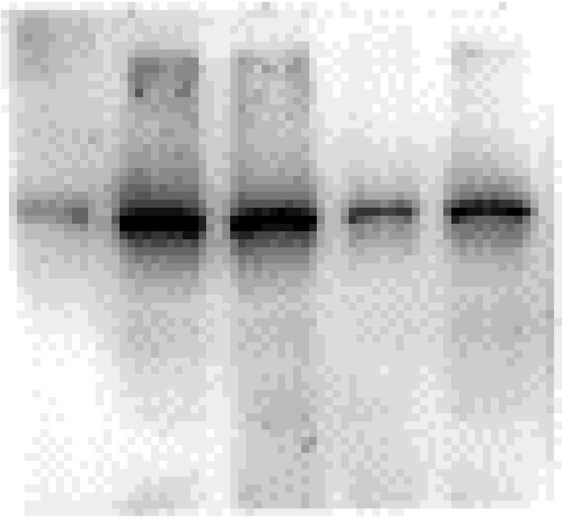

NFATc1

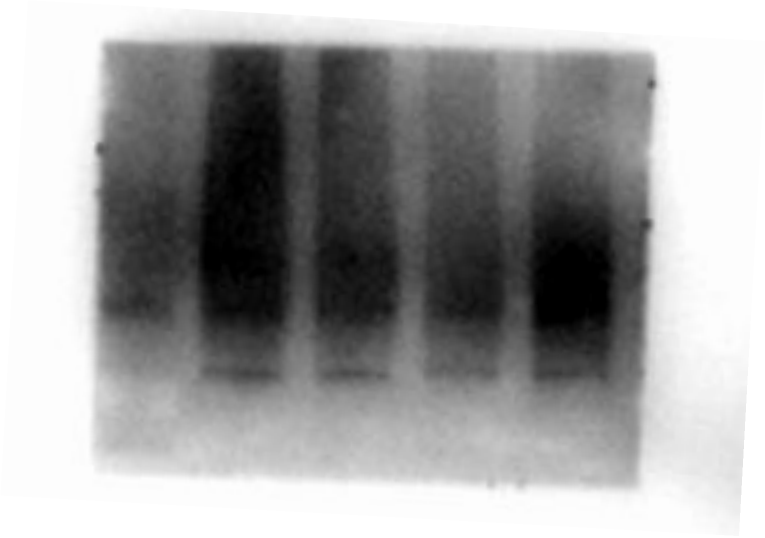

β-actin

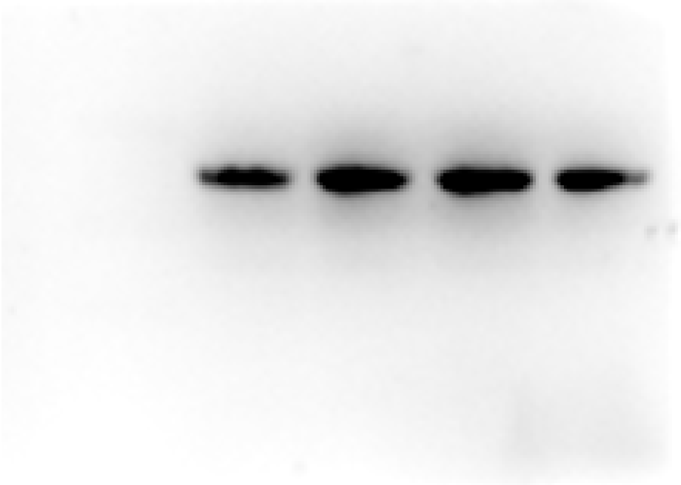

Blots from Fig4. E

MMP9

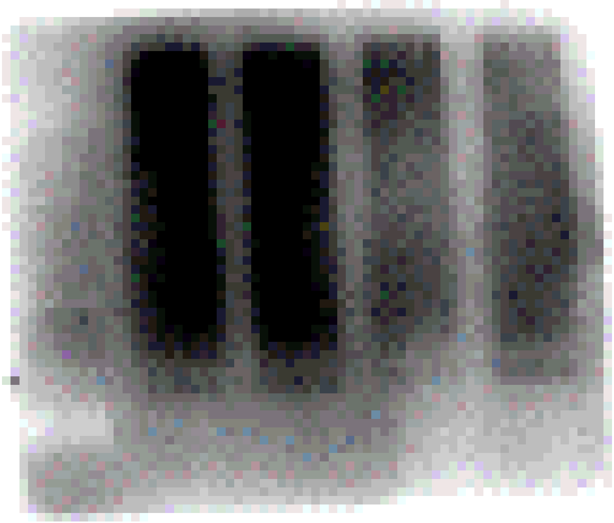

NFATc1

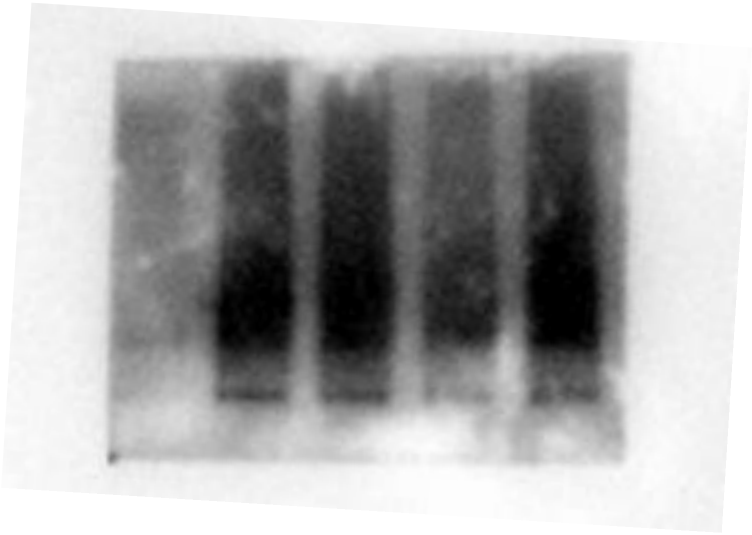

β-actin

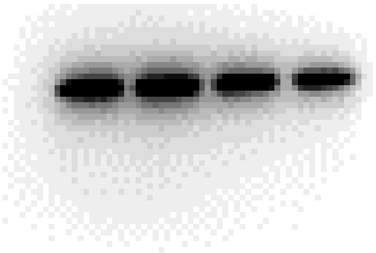

Blots from Fig5. I

PERK

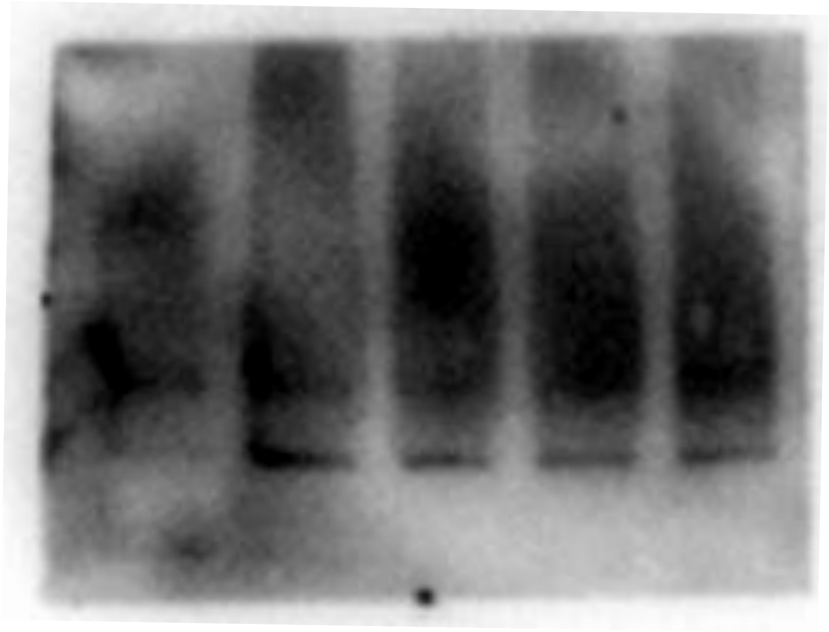

p-PERK

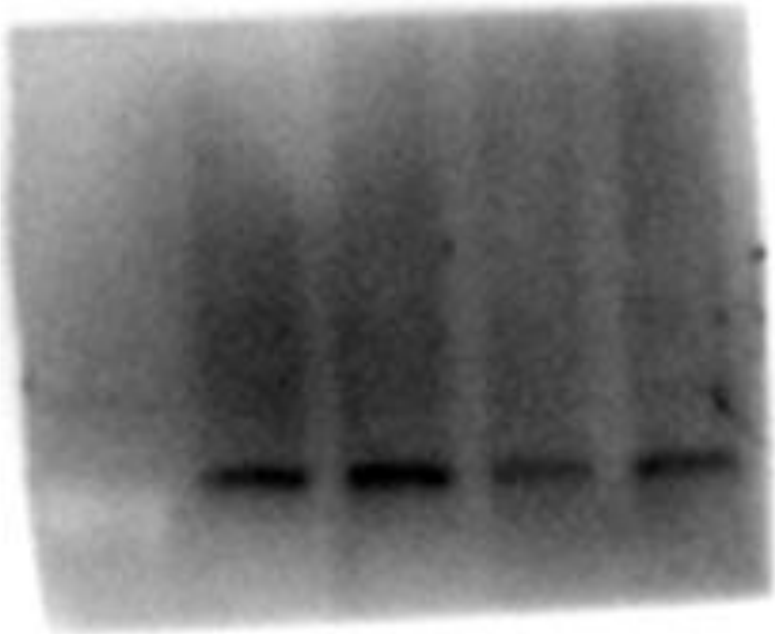

eIf2α

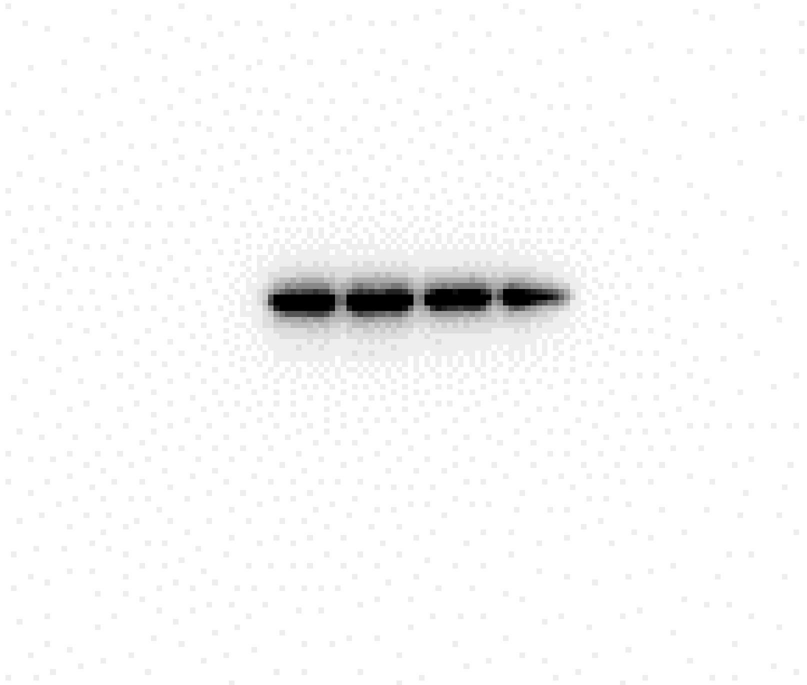

p-eIf2α

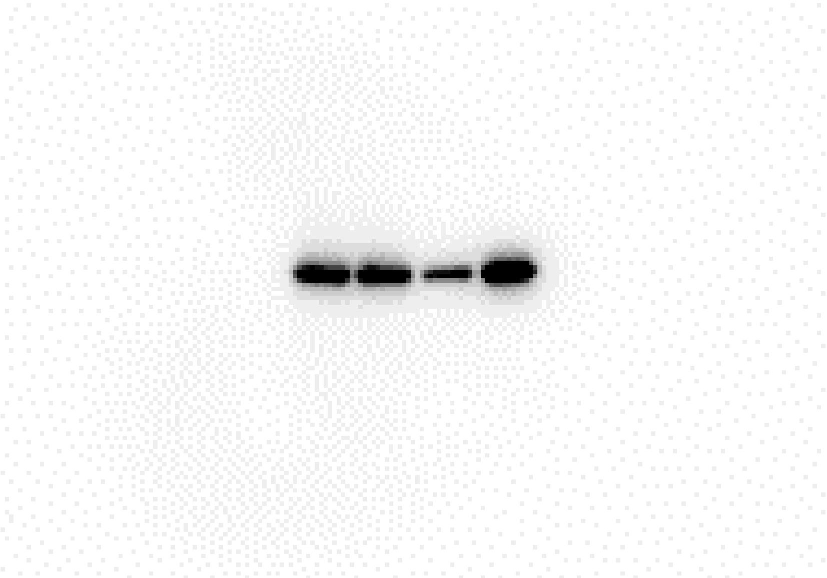

ATF4

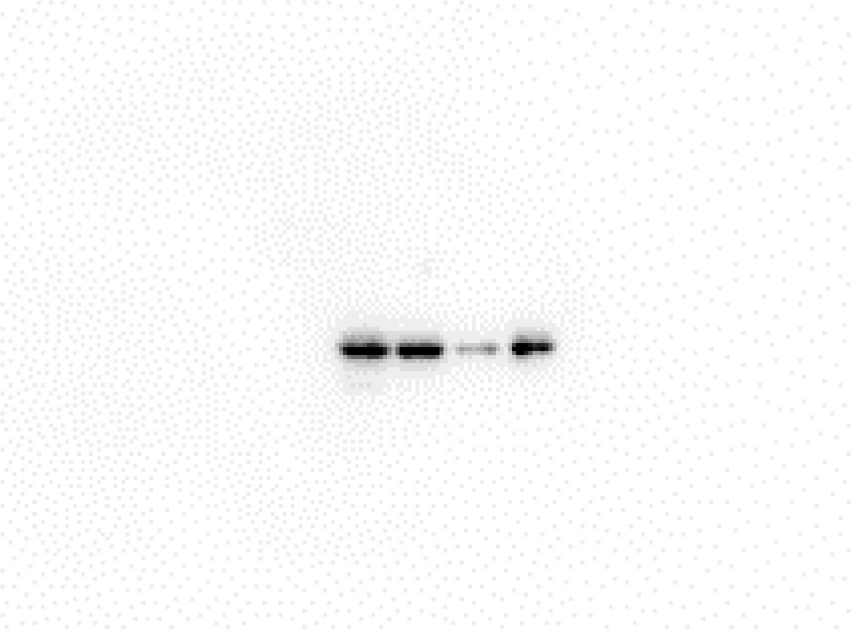

CHOP

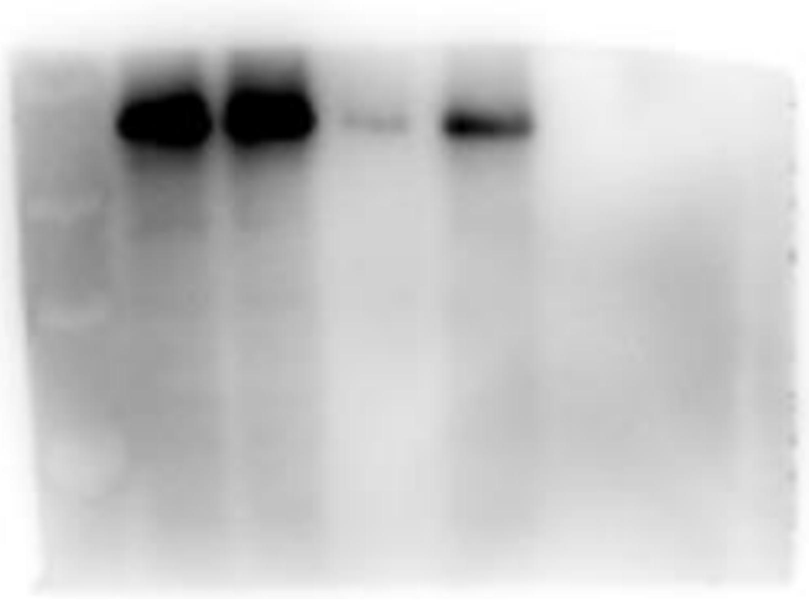

β-actin

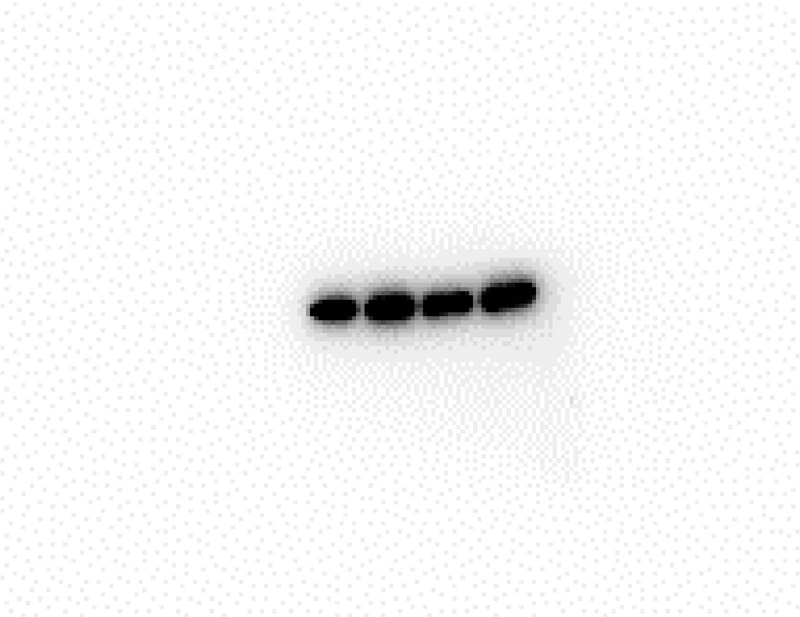

Blots from FigS1. A

TRPA1

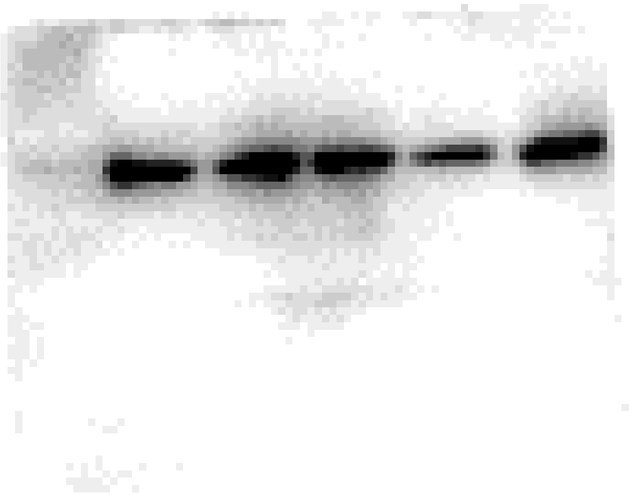

β-actin

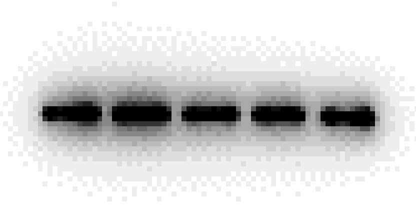

Blots from FigS1. C

PERK

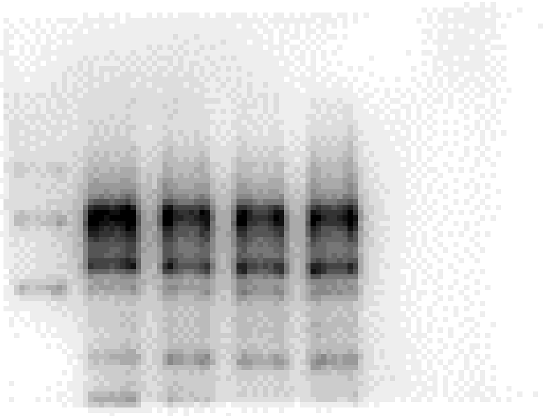

p-PERK

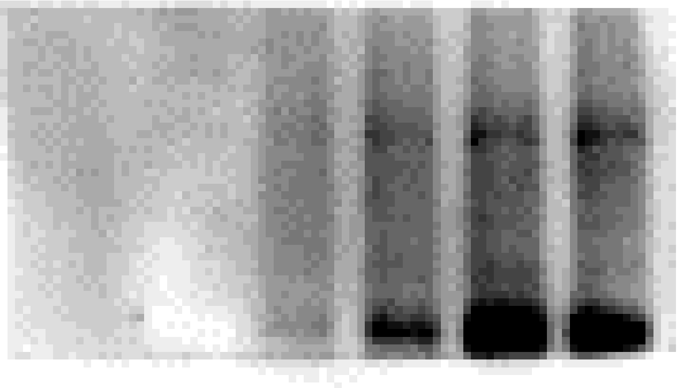

eIf2α

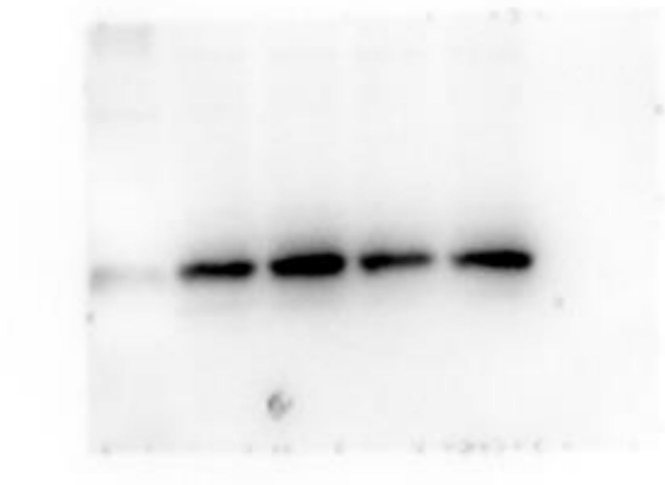

p-eIf2α

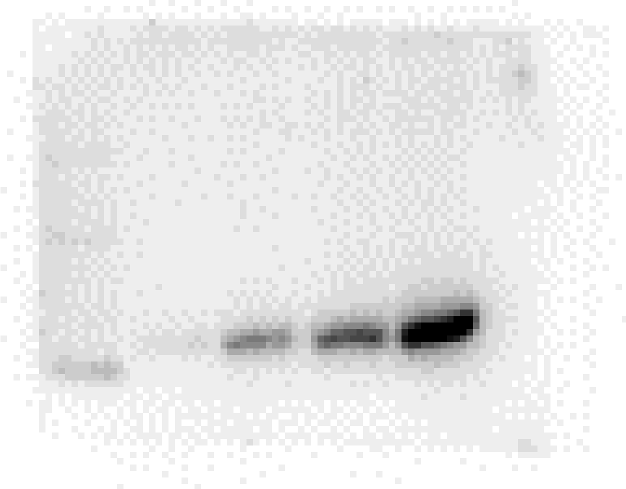

ATF4

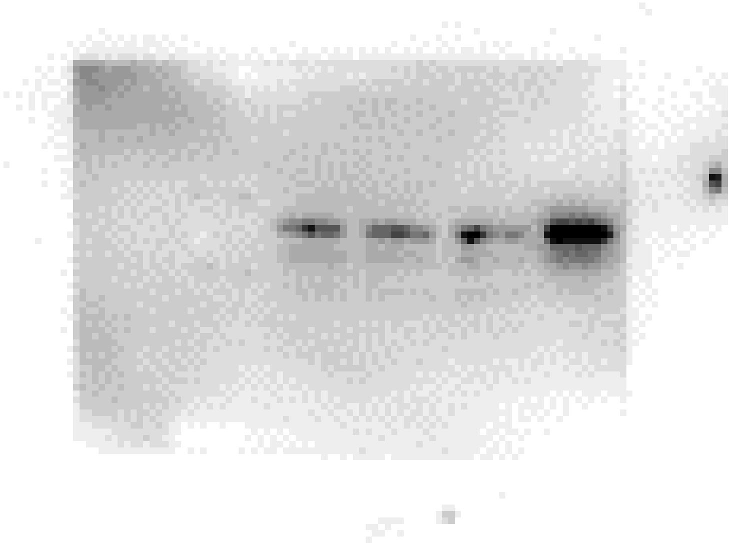

CHOP

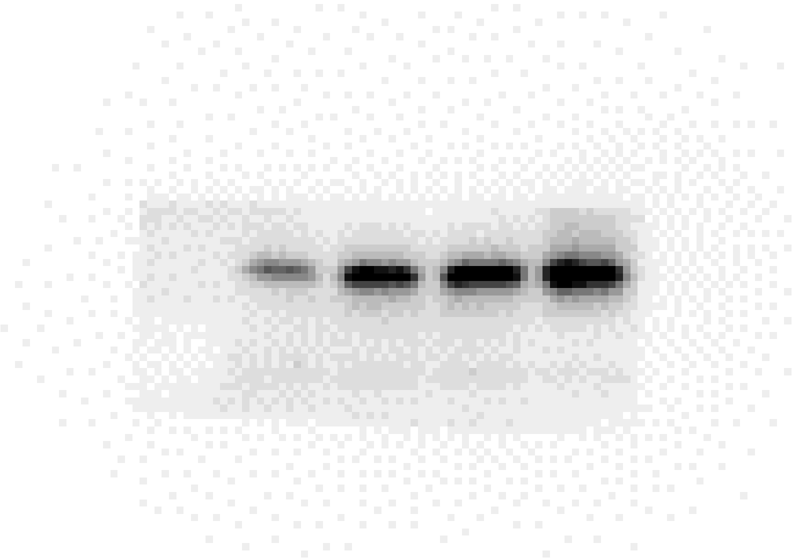

β-actin

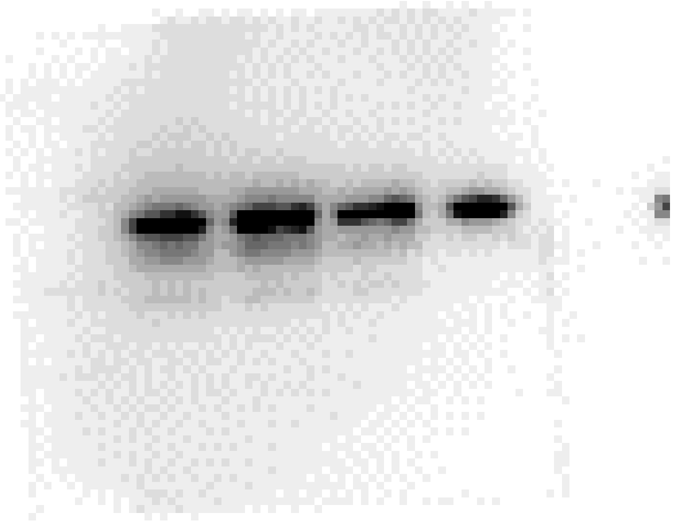

Blots from FigS2. E

TRPA1

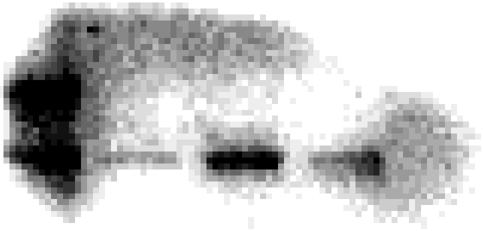

MMP9

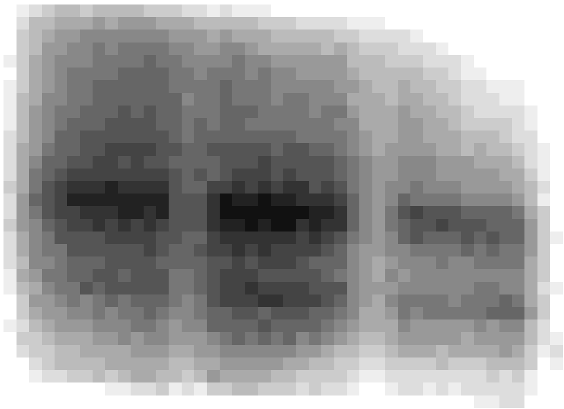

NFATc1

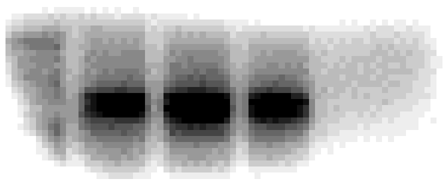

β-actin

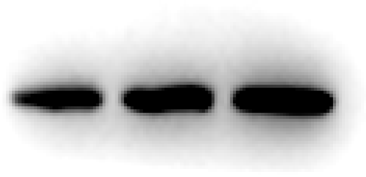

Supplement: Supplementary file 1 — Supplementary Information [file 41419_2024_7018_MOESM1_ESM.pdf]
